# Is the extrastriate body area part of the dorsal visuomotor stream?

**DOI:** 10.1007/s00429-017-1469-0

**Published:** 2017-07-12

**Authors:** Marius Zimmermann, Rogier B. Mars, Floris P. de Lange, Ivan Toni, Lennart Verhagen

**Affiliations:** 1 Donders Institute for Brain, Cognition and Behaviour, Radboud University, Nijmegen, The Netherlands; 2 Department of Psychology, Stockholm University, Stockholm, Sweden; 3 Wellcome Centre for Integrative Neuroimaging, Centre for Functional MRI of the Brain (FMRIB), Nuffield Department of Clinical Neurosciences, John Radcliffe Hospital, University of Oxford, Oxford, UK; 4 Department of Experimental Psychology, University of Oxford, Oxford, UK

**Keywords:** Category-selective visual areas, Ventral and dorsal visual pathways, Goal-directed action, Connectivity profile, Resting-state fMRI, Diffusion MRI

## Abstract

The extrastriate body area (EBA) processes visual information about body parts, and it is considered one among a series of category-specific perceptual modules distributed across the occipito-temporal cortex. However, recent evidence raises the possibility that EBA might also provide an interface between perception and action, linking the ventral and dorsal streams of visual information processing. Here, we assess anatomical evidence supporting this possibility. We localise EBA in individual subjects using a perceptual task and compare the characteristics of its functional and structural connectivity to those of two perceptual areas, the lateral occipital complex (LOC) and the fusiform body area (FBA), separately for each hemisphere. We apply complementary analyses of resting-state fMRI and diffusion-weighted MRI data in a group of healthy right-handed human subjects (*N* = 31). Functional and structural connectivity profiles indicate that EBA interacts more strongly with dorsal-stream regions compared to other portions of the occipito-temporal cortex involved in processing body parts (FBA) and object identification (LOC). These findings provide anatomical ground for a revision of the functional role of EBA. Building on a number of recent observations, we suggest that EBA contributes to planning goal-directed actions, possibly by specifying a desired postural configuration to parieto-frontal areas involved in computing movement parameters.

**Electronic supplementary material:**

The online version of this article (doi:10.1007/s00429-017-1469-0) contains supplementary material, which is available to authorized users.

## Introduction

The occipito-temporal cortex extracts information from early visual areas for further perceptual processing along the inferior temporal lobe. This cortical territory is thought to operate as a gateway for perceptual processing in the ventral visual stream and to remain largely separated from a dorsal visual stream that processes visual information relevant to motor control (Goodale and Milner 1992; but see Milner 2017). The extrastriate body area (EBA) has been regarded a case in point. This area responds selectively to images of the human body (Downing et al. 2001). It has been described as a purely perceptual area (Downing and Peelen 2011), processing visual information in a fashion similar to other category-specific regions in ventral occipito-temporal cortex such as the fusiform face area (FFA) (Kanwisher et al. 1997; Hutchison et al. 2014).

However, it has been suggested that EBA might also provide an interface between perceptual and motor processes (Astafiev et al. 2004; David et al. 2007; Gallivan et al. 2011; Kühn et al. 2011; Bracci et al. 2012; Tomasino et al. 2012; Limanowski et al. 2014; Orgs et al. 2016; Simos et al. 2017). Such an interface is required during goal-oriented behaviour that relies on perceptual knowledge, as when grasping a hammer according to its use. EBA could interface perceptual and motor processes in two ways. One possibility is that EBA’s contributions resemble that of the lateral occipital complex (LOC) (Malach et al. 1995). LOC provides access to perceptual information of object representations (James et al. 2003; Verhagen et al. 2008; Gallivan et al. 2015), and EBA might implement the same perceptual function for representations of body parts (Pitcher et al. 2009; Hutchison et al. 2014; Lingnau and Downing 2015). Another possibility is that EBA is more directly involved in motor control, specifying a desired postural configuration chosen from multiple possibilities during object manipulating actions (van Nuenen et al. 2012; Zimmermann et al. 2012, 2016). Here, in a strongly hypothesis-driven approach, we investigate the anatomical evidence to distinguish between the patterns of connectivity implied by those two possibilities.

Building on recent explorative whole-brain analyses of occipito-temporal connectivity at rest (Hutchison et al. 2014; Lingnau and Downing 2015) and investigations of EBA activity and connectivity in the context of task-related networks (e.g., Beer et al. 2013; Zimmermann et al. 2013; Orgs et al. 2016; Simos et al. 2017), we test whether EBA shows stronger functional and structural connectivity to the dorsal visuomotor stream than other areas of the occipito-temporal cortex that are sensitive to stimulus category. If the role of EBA is mainly perceptual, then this area is expected to have a connectivity profile similar to those of other portions of the ventral visual stream involved in processing body parts (i.e., the fusiform body area, FBA; Peelen and Downing 2005) and in identifying objects (i.e., the lateral occipital complex, LOC; Malach et al. 1995). If EBA directly contributes to planning goal-directed actions, then the connectional affinity of this area with dorsal-stream regions is expected to be stronger than that of either FBA or LOC. We distinguish between these two possibilities by considering two complementary indexes of connectivity, diffusion-weighted MRI (dw-MRI) and resting-state fMRI (rs-fMRI). Dw-MRI is a structural index of anatomical connectivity, ideally suited for non-invasive mapping of white-matter fibre systems, whereas resting-state fMRI is a functional index of (multi-synaptic) anatomical connectivity (O’Reilly et al. 2013), based on intrinsic coupled modulations in spontaneous activation between brain areas in the absence of external stimuli or task demands (Biswal et al. 1995; Fox and Raichle 2007; Hagmann et al. 2008; Honey et al. 2009). The strength and novelty of this study lie in combining data-driven and hypothesis-driven analyses of both functional and structural connectivities to make statements about EBA’s position within dorsal and ventral visual stream circuits (Passingham 2013).

## Methods

### Overview

The data used in this study were collected at the onset of a larger multi-session study (Zimmermann et al. 2016), but they have not been reported before.

### Participants

Thirty-two healthy, right-handed participants (25 ± 3 years, 17 male) gave written informed consent to take part in the study and were financially compensated at a rate of 10 euro/h. One subject was excluded from the resting-state analyses due to excessive head movements (>3 mm) during rs-fMRI data collection.

### MR scans and procedures

Each participant completed a series of four scans. All scans were completed within one session in a 1.5 T MR scanner (Avanto; Siemens, Erlangen, Germany) equipped with a 32-channel head coil for signal reception. Following an anatomical scan (T1-weighted MP-RAGE sequence, TR/TE = 2300/3.03 ms, voxel size 1 × 1 × 1 mm), participants performed a visual 1-back task that served as functional localizer for EBA, FBA, and LOC (see “Acquisition of functional localizer scans for EBA, FBA and LOC”), followed by resting-state and diffusion-weighted MRI scans (see “Acquisition of resting-state fMRI and diffusion-weighted MRI data”).

During all scans, subjects lay in supine position in the scanner. Cushions on each side of the head were used for stabilization. In addition, subtle tactile feedback about head movements was provided to the subjects by spanning tape from both sides of the head coil over the forehead, making it easier for them to minimize movements. A mirror construction attached to the head coil allowed participants to see a screen at the head end of the scanner bore, where stimuli could be presented.

### Acquisition of functional localizer scans for EBA, FBA, and LOC

Functional localization of EBA, FBA, and LOC was done using a 1-back task to enforce attention to the stimuli. Three sets of stimuli were used during this task. For localization of EBA and FBA, a set of 20 pictures of human bodies with digitally occluded heads was used; for LOC, we used a set of 20 pictures of man-made objects (e.g., keyboard, guitar, and window); the third set consisted of phase-scrambled versions of the 20 object pictures. Stimuli were presented in blocks of 20 stimuli + 2 stimuli repetitions that had to be detected by the participants (1-back task; 10 blocks per condition). Stimulus presentation time was 300 ms with a 450 ms inter-stimulus interval. Across trials, the location of the stimuli on the screen was randomly shifted (stimulus size: ~10° visual angle, shifted by 3.5° horizontally/vertically). Participants held a button box in their right hand and used their index finger to press a single button on the response box.

For the localizer scan, we acquired 256 whole-brain T2*-weighted multi-echo planar images [TR = 2180 ms, TE(1) = 9.4 ms, TE(2) = 21.2 ms, TE(3) = 33.0 ms, TE(4) = 45.0 ms; 31 slices, voxel-size 3.5 × 3.5 × 3.0 mm, gap-size 0.5 mm].

### Acquisition of resting-state fMRI and diffusion-weighted MRI data

Participants were instructed to keep awake with their eyes closed for the time of the resting-state scan, which lasted about 10 min. The dw-MRI scan took 9 min. The light in the scanner room was dimmed during both scans.

The resting-state scan consisted of 266 whole-brain T2*-weighted multi-echo planar images [TR = 2000 ms, TE(1) = 6.9 ms, TE(2) = 16.2 ms, TE(3) = 25.0 ms, TE(4) = 35 ms, TE(5) = 44 ms; 39 slices, voxel-size 3.5 × 3.5 × 3.0 mm, gap-size 0.5 mm]. Diffusion-weighted data were acquired using echo planar imaging (64 2.2 mm-thick axial slices; field of view 220 × 220 mm; voxel size 2.2 × 2.2 × 2.2 mm). Diffusion weighting was isotropically distributed along 61 directions using a *b* value of 1000 s/mm^2^. Seven volumes with no diffusion weighting were acquired throughout the acquisition.

### Image preprocessing and analysis of functional MRI data

All functional images, for the localizer task as well as the resting-state scan, were analysed using MATLAB (R2009b; MathWorks, Natick, MA, USA) and SPM8 (Wellcome Department of Cognitive Neurology, London, UK). Regions of interest (ROI) masks were constructed using MarsBaR (Brett et al. 2002).

First, functional images were spatially realigned using a least-squares approach that estimates rigid-body transformations (translations and rotations) by minimizing head movements between the first echo of each image and the reference image (Friston et al. 1995a). Next, all echoes of one image were combined into a single volume. For this, the first 30 volumes of each timeseries (functional localizer scan or resting-state scan) were used to estimate the best weighted echo combination to optimally capture the BOLD response over the brain (Poser et al. 2006). These weights were then applied to the entire timeseries. Subsequently, the timeseries for each voxel were temporally realigned to the acquisition of the first slice. Anatomical images were spatially coregistered to the means of the functional images. Normalization parameters to transform anatomical images to a standard EPI template centred in MNI space (Ashburner and Friston 1999) were estimated and used to transform individual structural and functional images into a standard space for group analyses, with a voxel size of 2 × 2 × 2 mm. Finally, images were smoothed with a 6 mm full-width at half-maximum (FWHM) kernel.

For the localizer task, square-wave functions corresponding to the block duration were constructed for each of the three image categories (bodies, objects, and scrambles), and convolved with a canonical haemodynamic response function and its temporal derivative (Friston et al. 1995b). In addition, the statistical model included 13 separate regressors of no interest, modelling button presses, and residual head movement-related effects by including the six rigid-body motion parameters (translations and rotations), as well as their first-order temporal derivatives. Parameter estimates for all regressors were obtained by maximum-likelihood estimation, using a temporal high-pass filter (cutoff 128 s), modelling temporal autocorrelation as a first-order autoregressive process. Linear contrasts pertaining to the main effects of the design were calculated.

EBA and FBA were identified by comparing statistical parametric maps of the ‘body’ condition with those of the ‘object’ condition, providing locations for left and right EBA and FBA (Downing et al. 2001; Peelen and Downing 2005). LOC was identified by comparing statistical parametric maps of the ‘object’ condition with those of the ‘scrambled’ condition, providing locations for left and right LOC (Malach et al. 1995). For each ROI, we identified the most significantly activated voxel within a restricted area of the cortex, which was based on previously published locations (EBA: Downing et al. 2001; FBA: Peelen and Downing 2005; LOC: Malach et al. 1995).

Using individual locations for left and right EBA, FBA, and LOC, timeseries for the seed regions were extracted from the resting-state data for each participant. Separate GLMs were constructed for each of the six seed regions (l/rEBA, l/rFBA, and l/rLOC). Each GLM included the first eigenvalue timeseries of a 4 mm sphere around the individuals’ peak coordinates for the seed region. 15 additional regressors were included in each design matrix, modelling residual head movement effects by including the six rigid-body motion parameters (translations and rotations), as well as their first temporal derivatives, and compartment signals for white matter, cerebro-spinal fluid, and out-of-brain signals (Verhagen et al. 2008). Parameter estimates for the connectivity between the seed region and the rest of the brain were obtained by maximum-likelihood estimation, using a temporal high-pass filter (cutoff 128 s) and modelling temporal autocorrelation as a first-order autoregressive process. Estimated beta-maps related to the seed regions’ time-course regressors were used for subsequent analyses (see below).

### Functional connectivity analyses: overview

First, we characterized the connectivity patterns for the different seed regions EBA, FBA, and LOC in a descriptive analysis to identify connected regions at the whole-brain level (“Part I: explorative analyses”). Differences and similarities between these patterns were illustrated using connectivity fingerprints. Subsequently, we quantified differences between these patterns in two complementary statistical analyses (“Part II: hypothesis-driven analyses”). Differences in connectivity strength with dorsal and ventral stream regions were quantified using ROI-based analyses (“ROI-based functional connectivity”). Differences between connectivity patterns of EBA, FBA, and LOC were quantified using a multivariate classifier trained on whole-brain connectivity patterns of the superior parietal and inferior temporal lobules (“Seed-region classification on whole-brain connectivity patterns”).

#### Part I: explorative analyses

##### Estimating whole-brain connectivity patterns and contrasts

To describe the connectivity pattern of EBA, FBA, and LOC, we identified areas whose timeseries were correlated with those of the seed regions at the whole-brain level. In SPM, we conducted a within-subject analysis of the beta-images obtained during the first-level analysis (see “Image preprocessing and analysis of functional MRI data”), treating participants as a random factor. In addition, we contrasted the connectivity patterns to identify the areas that are differently co-activated with the seed regions, in both directions (e.g., EBA > LOC, LOC > EBA). Both whole-brain analyses were performed using a two-step procedure, where first clusters were formed using a cluster-forming threshold of *p* < 0.001 (uncorrected), followed by identification of significant clusters at *p* < 0.05 (FWE corrected). Where possible, activated clusters were assigned an anatomical label using SPM’s Anatomy toolbox (Eickhoff et al. 2005).

##### Creation of connectivity fingerprints

To further describe connectivity profiles for the seed regions, we established ‘fingerprints’ based on coupling with a set of 13 ipsilateral target ROIs in MNI space (Table 1). The purpose of the fingerprints was to simplify visualisation of the whole-brain connectivity maps, i.e., the similarities and differences between the maps of the different seed regions. The target regions were a relevant sample selected a posteriori from the whole-brain connectivity maps and contrast maps between seed regions, choosing regions that would add most value to visualize similarities and differences between connectivity profiles. Regions were centred on peaks in the statistical parametric maps. Each target region consisted of a sphere with 4 mm radius, equivalent to 33 voxels, created with MarsBaR and SPM8. Per subject and localizer ROI seed region, average beta values of the target ROIs were extracted and summarized over participants. Values were masked at zero, following procedures used by Mars and colleagues (2011) (Sallet et al. 2013; Neubert et al. 2014). Spider-plot diagrams were created, illustrating the connectivity fingerprint of each seed region.

**Table 1:** List of target regions and MNI coordinates used for connectivity fingerprints (Fig. 4)

Label	Region	MNI		
		*X*	*Y*	*Z*
BA45	Brodmann area 45	±54	+24	+16
BA2	Brodmann area 2	±42	−30	+48
OP	Parietal operculum	±54	−18	+18
BA5	Brodmann area 5	±18	−52	+62
BA7a	Brodmann area 7 (anterior)	±32	−43	+58
V1	Primary visual cortex	±8	−90	+2
FFG	Fusiform gyrus	±42	−50	−8
IT	Inferior temporal lobe	±35	−36	−20
MTS	Medial temporal sulcus	±58	−36	−4
STS	Superior temporal sulcus	±58	−28	+14
Ins	Insula	±52	+4	−14
Hipp	Hippocampus	±20	−34	−4
OFC	Orbito-frontal cortex	±4	+58	−14

We quantified the similarities and differences between fingerprints using connectivity fingerprint matching (Mars et al. 2016b). Permutation testing (Nichols and Holmes 2002) was used to test the significance of the difference between seed regions within each hemisphere and between homologues across hemispheres. We tested the hypothesis that the difference between regions’ connectivity fingerprints, as indexed by the city-block distance (i.e., the sum of differences over all fingerprint arms between a pair of regions), is larger than expected by chance. To obtain a robust estimate of the chance level, we calculated the city-block distance for each relevant pair of fingerprints for 5000 different permutations of the seed-region labels. For each test, the fingerprints were normalized to a range between 0 (weakest connection with any of the target regions) and 1 (strongest connection with any of the target regions). In order not to bias the test, the normalization was performed over the combined arms of both fingerprints. Subsequently, we used logistic regression to assess which target regions significantly contributed to observed differences between hemispheric homologues. Given that these analyses served to further illustrate the whole-brain analyses, these statistical analyses were not corrected for multiple comparisons.

#### Part II: hypothesis-driven analyses

##### Definition of dorsal and ventral stream target regions

In the following analyses, we investigate the connectivity between the seed regions and regions of interest representing the core of the dorsal and ventral stream. Following Goodale and Milner (1992), Milner and Goodale (2008), for the purposes of the current study, we focus on the ‘dorsal vision-for-action’ stream along the lateral superior and inferior parietal lobes, ‘dorsal stream’ in short. Similarly, we focus on the ‘ventral vision-for-identification’ processing stream along the inferior temporal lobe, ‘ventral stream’ in short. Dorsal and ventral stream ROIs were based on sets of the cortical parcellation of Glasser and colleagues using multi-modal analyses of magnetic resonance images from the Human Connectome Project (HCP-MMP1.0 atlas, Glasser et al. 2016). The choice of regions that constitute our dorsal and ventral stream regions of interest was based on a review of existing literature on the two streams (Mishkin and Ungerleider 1982; Felleman and Van Essen 1991; Goodale and Milner 1992; Young 1992; Milner and Goodale 2008; Kravitz et al. 2011, 2013) and on a description of the HCP-MMP1.0 atlas (Glasser et al. 2016). In short, we have aimed to select regions with a consistent functional profile adhering to our hypothesis while being both generously inclusive to avoid selection bias and restrictive, where proximity bias might otherwise potentially skew the results. Specifically, for the dorsal-stream region of interest, we included lateral regions of the posterior parietal cortex, spanning both the superior and inferior parietal lobe (avoiding selection bias). We excluded occipital areas to adhere to our hypothesis focussed on ‘vision-for-action’ (Young 1992) and to avoid potentially artificially strong connectivity of EBA with our target ROI due to proximity. Similarly, we excluded parietal regions that constituted clearly distinct functional processing circuits with characteristic connectional profiles, both along the medial wall (Kravitz et al. 2011) and belonging to the temporoparietal junction (TPJ; Mars et al. 2011). For the ventral stream region of interest, we focussed on the whole inferior temporal lobe while excluding several bordering areas following the same criteria as for the dorsal-stream definition. Namely, we excluded occipital regions to avoid proximity bias, and areas in lateral and superior temporal cortex based on their distinctive functional and connectional profiles (including connections with the dorsal stream; Kravitz et al. 2013). The selected regions are listed in Table 2. To obtain individuated regions of interest, we processed each participant’s T1-weighted anatomical image according to the open-source HCP Minimal Processing Pipeline (Glasser et al. 2013), or more specifically, according to a version of the Pipeline that does not require a T2-weighted image (https://github.com/lennartverhagen/Pipelines). This allowed us to directly map the HCP-MMP1.0 atlas from the average cortical surface back to a participant’s individual volumetric MR image for further statistical analysis.

**Table 2:** List of regions selected from the HCP-MMP1.0 atlas (Glasser et al. 2016) forming part of dorsal and ventral stream ROIs

Area name^a^	Area description^a^	Stream
TE1a	Area TE1 anterior	Ventral
TE1p	Area TE1 posterior	Ventral
TE2a	Area TE2 anterior	Ventral
TF	Area TF	Ventral
TE2p	Area TE2 posterior	Ventral
PHT	Area PHT	Ventral
PH	Area PH	Ventral
TGv	Area TG ventral	Ventral
TE1 m	Area TE1 middle	Ventral
IPS1	Intraparietal sulcus area 1	Dorsal
7AL	Lateral area 7A	Dorsal
7PI	Lateral area 7P	Dorsal
7PC	Area 7PC	Dorsal
LIPv	Area lateral intraparietal ventral	Dorsal
VIP	Ventral intraparietal complex	Dorsal
MIP	Medial intraparietal complex	Dorsal
PFt	Area PFt	Dorsal
IP2	Area intraparietal 2	Dorsal
IP1	Area intraparietal 1	Dorsal
PF	Area PF complex	Dorsal
PFm	Area PFm complex	Dorsal
V6A	Area V6A	Dorsal

^a^Area name and area description referring to labels as used in Glasser et al. (2016)

##### ROI-based functional connectivity

We were interested in assessing the connectivity profile of EBA in relation to those of prototypical perceptual regions such as FBA and LOC. This was tested by investigating the connectivity of the seed regions (EBA, FBA, and LOC) to sets of dorsal and ventral stream regions (see “Definition of dorsal and ventral stream target-regions”).

Using Matlab, we extracted the average connectivity strength (beta values) of the seed regions (EBA and LOC) with the two ipsilateral target regions (dorsal and ventral). Two-way ANOVAs with factors seed region (EBA, FBA, and LOC) and target region (dorsal and ventral) were used to test for significant interactions between seed- and target regions within each hemisphere. A three-way ANOVA with the additional factor hemisphere (left and right) was used to test for potential differences in hemispheric specialization. For all ROI-based analyses, values of *p* < 0.05 were considered statistically significant. In addition, we analysed the connectivity strength of the seed regions with each region of the HCP-MMP1.0 atlas (Glasser et al. 2016) individually. The aim of this analysis was to investigate whether any differences in connectivity strength with dorsal and ventral stream target regions are consistent over a wider range of regions and, therefore, robust against minor changes of our definition of dorsal and ventral stream target regions. The results of this analysis are presented in the supplementary material (Figure S3).

##### Seed-region classification on whole-brain connectivity patterns

Complementary to the above assessment of connectivity strength, we aimed to classify the connectivity patterns of the seed regions (EBA, FBA, and LOC) as resembling either a ‘dorsal’ or a ‘ventral’ profile. First, for each of the parcellation areas that together form our dorsal and ventral stream ROIs (see “Definition of dorsal and ventral stream target-regions”; Table 2), we calculated the whole-brain connectivity pattern in SPM8, using the same procedure as we used for the connectivity maps of the seed regions EBA, LOC, and FBA. The statistical parametric maps (beta-images) were labelled as dorsal or ventral according to Table 2, and formed the training set for a *k*-nearest-neighbour classifier in Matab (*knnclassify*; distance metric: city block). The number of nearest neighbours (*k*) was modulated from 2 to 8 (i.e., 1 less than the number of sub-regions in the ventral stream). This classifier was then used to classify the connectivity maps (beta-images) of the three seed regions (EBA, LOC, and FBA) for each participant. The outcome of the seed-regions’ classification was compared using a Friedman’s test with factors seed regions (EBA, LOC, and FBA) and *k* [2…8]. Friedman’s test is a non-parametric version of a balanced two-way ANOVA, which allows for testing of within-subject differences. A three-way ANOVA with the additional factor hemisphere (left and right) was used to test for potential differences in hemispheric specialization. For classification analyses, values of *p* < 0.05 were considered statistically significant.

##### Image processing and analysis of diffusion-weighted MRI

Analyses of diffusion-weighted images were performed using tools from FDT v2.0 (FMRIB’s Diffusion Toolbox) as part of FSL v5.0 (FMRIB's Software Library) and custom-made software written in Matlab. Eddy-current distortions were corrected using affine registration of all volumes to a target volume with no diffusion weighting. Voxelwise estimates of the fibre orientation distribution were calculated using BedpostX, limited to estimating two fibre orientations at each voxel, because of the *b* value and number of gradient orientations in the diffusion data (Behrens et al. 2007).

For each participant, the T1 image was linearly registered to the diffusion images (based on an image with no diffusion weighting as a target) using FLIRT (FMRIB’s Linear Image Registration Tool) and segmented using FAST (FMRIB’s Automated Segmentation Tool). The T1 image was non-linearly warped to the standard MNI space (as defined by the MNI152 template brain provided by FSL) using FNIRT (FMRIB’s Non-Linear Image Registration Tool). The resulting transformations were used to register the individual locations for EBA, FBA, and LOC to each participant’s diffusion space. Probabilistic tractography was run from all voxels in the sheet of white-matter voxels bordering the grey matter of our regions of interest (as obtained from the HCP Minimal Processing Pipeline). We seeded from the 20 white-matter voxels on the border with grey matter closest to each participant’s EBA, FBA, and LOC locations. We included only paths that terminated in the white-matter bordering the dorsal and ventral masks (see “Definition of dorsal and ventral stream target-regions”). We operationalized anatomical connectivity strength as the average of the log-transformed and normalized number of paths that originate from either EBA, FBA, or LOC and reach the dorsal and ventral target regions (Mars et al. 2016a). For analyses of diffusion-weighted MRI data, values of *p* < 0.05 were considered statistically significant. In addition, we analysed anatomical connectivity strength of the seed regions with each region of the HCP-MMP1.0 atlas (Glasser et al. 2016) individually. Again, the aim of this analysis was to investigate whether any differences in anatomical connectivity with dorsal and ventral stream target regions are consistent over a wider range of regions and, therefore, robust against minor changes of our definition of dorsal and ventral stream target regions. The results of this analysis are presented in the supplementary material (Figure S4).

## Results

### Seed regions EBA, FBA, and LOC

**Fig. 1:**
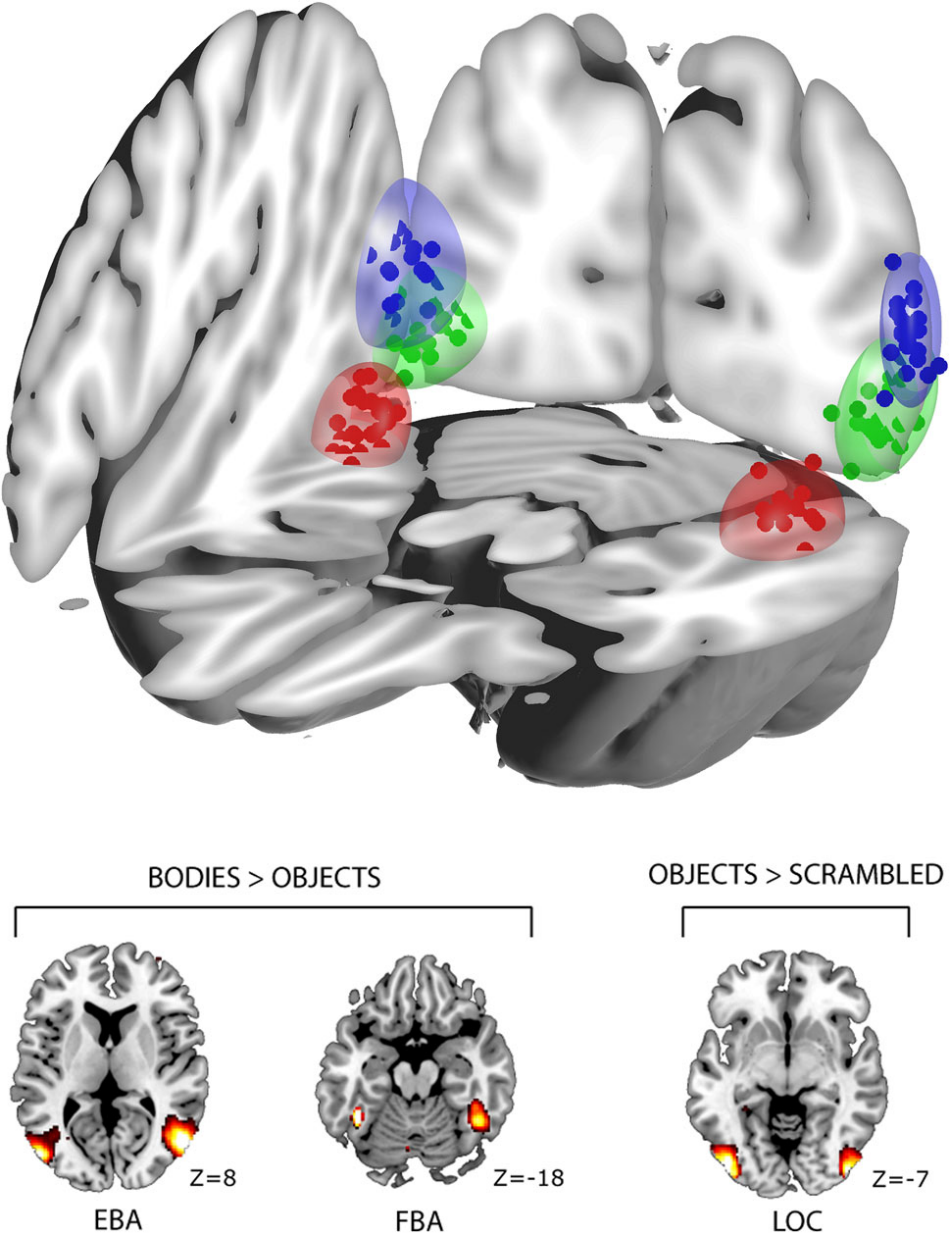
*Top* locations of subject-specific seed regions in EBA (*blue*), LOC (*green*), and FBA (*red*). *Ellipsoids* represent 95% confidence intervals in all three directions of each ROI location. A control analysis confirmed that there was no overlap between seed regions for any of the participants. *Bottom* seed-region locations and contrasts for functional localizer EBA, FBA (bodies > objects, *p*_(FWE)_ < 0.05; Downing et al. 2001; Peelen and Downing 2005) and LOC (objects > scrambled; Malach et al. 1995)

Average MNI coordinates for left and right EBA were [−49 −76 8] and [50 −73 4], for FBA [−41 −47 −18] and [42 −50 −17], and for LOC [−44 −80 −7] and [45 −79 −7]. The average distances between regions differed [left: *F*(2,92) = 172.94, *p* < 0.001; right: *F*(2,92) = 77.9, *p* < 0.001]; the distance between EBA and LOC was on average 17.9 ± 4.1 mm (left) and 17.1 ± 4.7 mm (right), between EBA and FBA, it was 41.5 ± 5.1 mm (left) and 33.4 ± 6.6 mm (right), and between LOC and FBA, it was 34.9 ± 6.3 mm (left) and 31.6 ± 5.5 mm (right) (Fig. 1). A control analysis confirmed that there was no overlap (*0 voxels*) between the seed regions (EBA, FBA, and LOC) for any of the participants.

#### Part I: explorative analyses

##### Whole-brain connectivity patterns

**Fig. 2:**
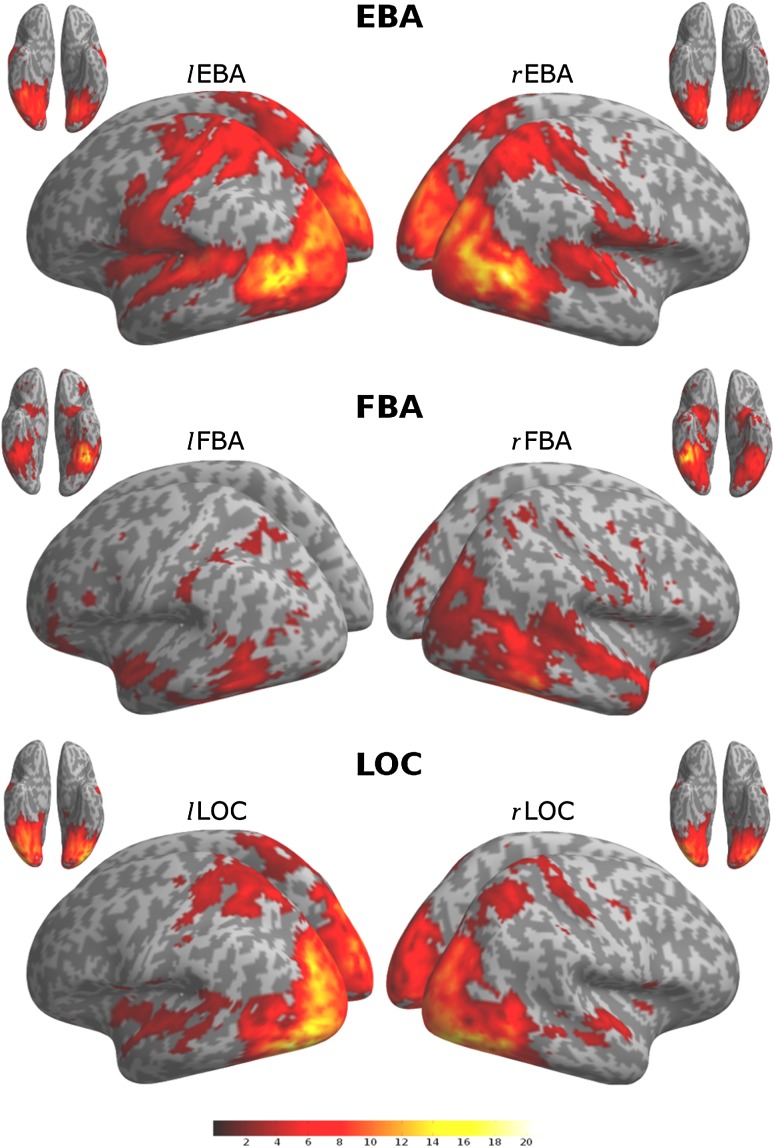
Whole-brain connectivity patterns for EBA (*top*), FBA (*middle*), and LOC (*bottom*), and left and right hemisphere seed regions. Clusters larger than 50 contiguous voxels are shown, on the basis of a cluster-forming threshold of *p* < 0.001. *Colours* and *colour bar* (*red–yellow*) indicate voxelwise *t* values

**Fig. 3:**
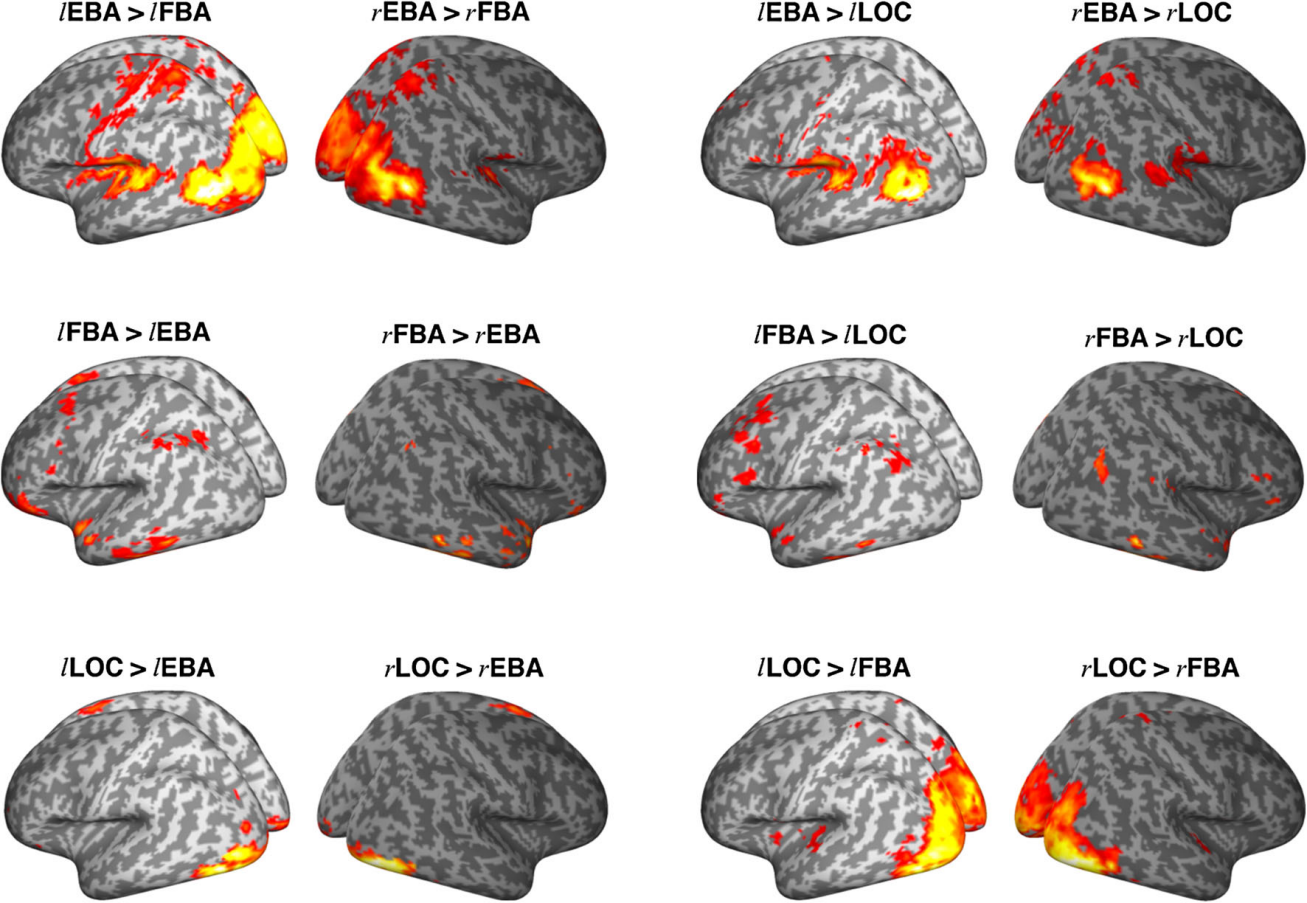
Contrast images of whole-brain connectivity patterns for left and right seed regions between EBA, FBA, and LOC. Clusters larger than 50 contiguous voxels are shown, on the basis of a cluster-forming threshold of *p* < 0.001. *Colours* (*red–yellow*) indicate voxelwise *t* values, for scale see Fig. 2. For a list of covered regions, see supplementary material (Table S1)

**Fig. 4:**
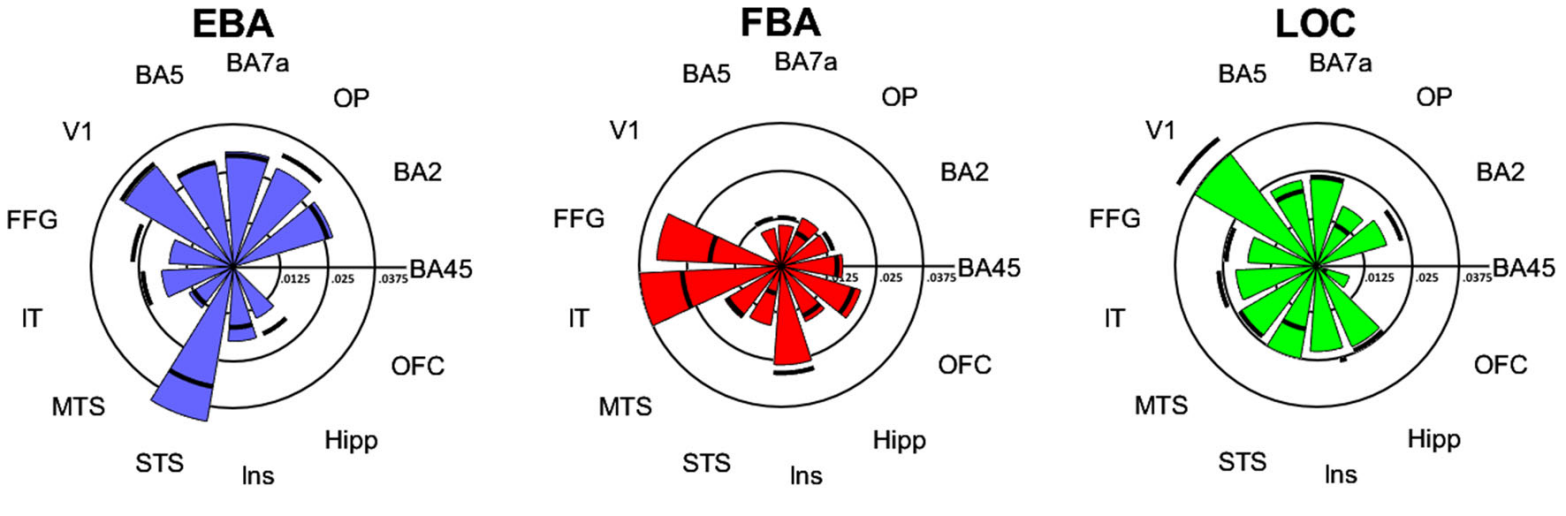
Connectivity fingerprints of seed regions EBA, FBA, and LOC for left (*wedges*) and right (*lines*) hemispheres. Only positive connections are shown. The *scale* indicates beta values. *BA* Brodmann area, *OP* operculum, *STS* superior temporal sulcus, *MTS* medial temporal sulcus, *IT* inferior temporal sulcus, *FFG* fusiform gyrus, *OFC* orbito-frontal cortex, *Hipp* hippocampus, *Ins* insula

**Fig. 5:**
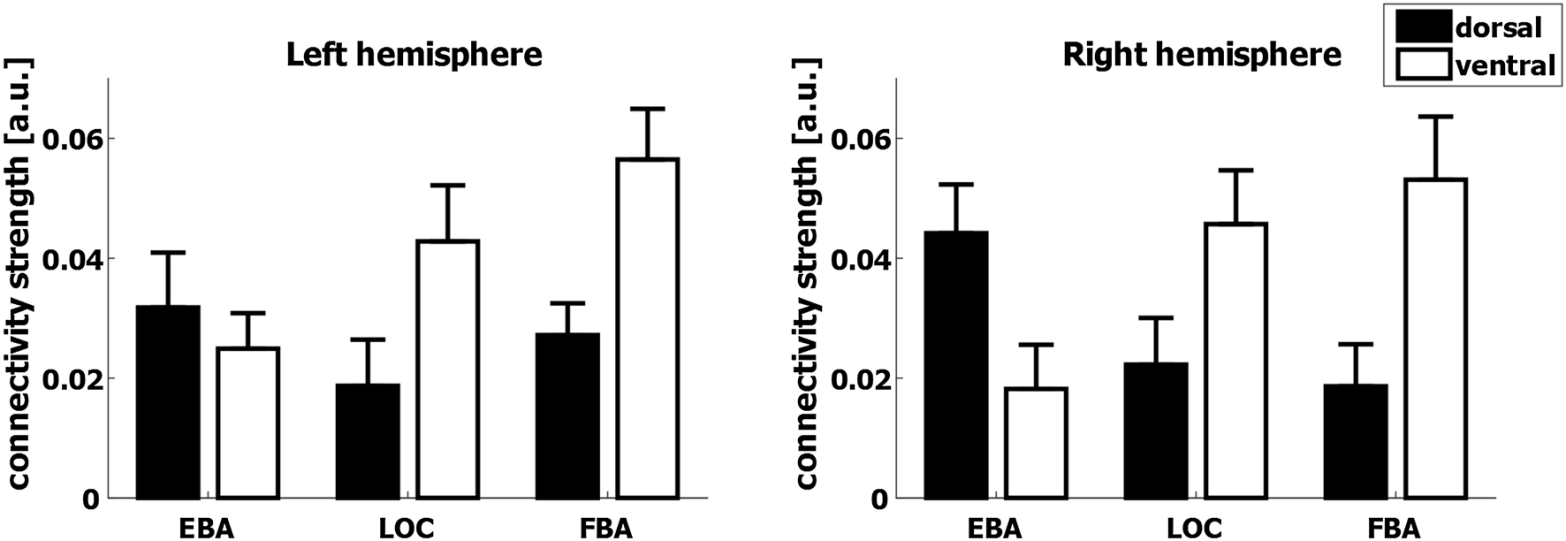
Resting-state connectivity strength (beta values) between seed regions (EBA, FBA, and LOC) and target regions in the dorsal visual stream (in *black*) or in the ventral visual stream (in *white*). In both hemispheres, EBA shows relatively stronger connectivity to the dorsal stream compared to LOC and FBA, which both show relatively stronger connectivity with the ventral stream. *Error bars* indicate the standard error of the mean

We used multiple regression analysis to identify brain regions where BOLD fluctuations were uniquely coupled to those of EBA, FBA, or LOC (Fig. 2). EBA and LOC functional connectivity maps showed large similarities, each sharing unique fluctuations with the occipital cortices, superior temporal lobes, superior parietal lobes, and post- and pre-central regions. FBA was coupled with the inferior temporal and occipital lobe and parts of the parietal cortex. These descriptive results replicate earlier findings (Hutchison et al. 2014), opening the way for a novel quantitative test of differences in the connectivity profiles of EBA, FBA, and LOC (Figs. 4, 5). As shown in Fig. 3, and fully reported in the supplementary materials (Table S1), we observed stronger interactions of EBA, compared to LOC, with left and right parietal operculum as well as parts of the mid-superior temporal gyrus. LOC interacts more strongly with areas around the posterior fusiform gyrus and inferior occipital and temporal cortex. EBA, compared to FBA, interacts more strongly with mid-superior temporal and occipital cortices, as well as postcentral regions. EBA interacts more strongly with the pre-central gyrus than either LOC or FBA. FBA, on the other hand, has stronger interactions with the inferior temporal gyrus, fusiform gyrus, parahippocampal gyrus, and pre-SMA. FBA, compared with LOC, interacts more strongly with inferior occipito-temporal cortex, anterior fusiform gyrus, and the left hippocampal area. LOC has stronger interactions with large parts of the occipital cortex, compared to FBA. In this exploratory analysis, we did not observe hemispheric specialization in the differences between connectivity patterns of seed regions (Fig. 3).

##### Connectivity fingerprints

To further characterize the connectivity patterns of EBA, FBA, and LOC, we represented the connectivity fingerprints in relation to a set of target regions in a spider plot (Fig. 4) that highlights differences in connectivity fingerprints between EBA, FBA, and LOC, and between left and right homologues. Permutation testing confirmed the exploratory whole-brain results and revealed that each region has an identifiable connectivity fingerprint (all comparisons between pairs of fingerprints *p* < 0.05, except rLOC-rFBA: *p* = 0.06; uncorrected). These fingerprints could potentially be used to identify these regions based on resting-state fMRI data alone, in the absence of localizer tasks (Saygin et al. 2012; Mars et al. 2013; Tavor et al. 2016; see also Osher et al. 2016). EBA is characterized by connections with the superior temporal sulcus and with parietal regions involved in motor control, including BA5 (SPL), BA7a (anterior IPS), OP, and BA2, which are weaker for the other seed regions. The connectivity fingerprint for FBA is characterized by its strong connections with the fusiform gyrus and the inferior temporal lobe. The connectivity fingerprint of LOC is less biased towards connections with either dorsal or ventral stream regions. Finally, only EBA and LOC, but not FBA, connect strongly with primary visual areas. When testing for specialization between hemispheric homologues of these regions, the connectivity fingerprints emphasized differences between left and right EBA (*p* = 0.03), but not FBA (*p* = 0.19) and LOC (*p* = 0.18). Logistic regression revealed that the hemispheric specialization of EBA was mostly driven by stronger connectivity of right than left EBA with OP (*p* = 0.04), STS (*p* = 0.01), and FFG (*p* = 0.04), but notably not by BA5 (*p* = 0.98), BA7a (*p* = 0.69), and BA2 (*p* = 0.44). This suggests that both left and right EBA were strongly connected with superior parietal regions, but differed in their coupling with temporal regions.

#### Part II: hypothesis-driven analyses

##### ROI-based functional connectivity

After characterizing the connectivity fingerprints, we directly tested whether EBA would show stronger connectivity strength with the dorsal stream and is more likely to resemble a dorsal-stream connectivity profile, as compared to FBA and LOC (Fig. 5). First, we assessed the connectivity strength of EBA, FBA, and LOC with the dorsal and ventral streams. Using large ROIs spanning ventral and dorsal-stream regions based on the whole-brain cortical parcellation atlas (HCP-MMP1.0, Glasser et al. 2016; see “Methods” for details), a 3 × 2 ANOVA with factors seeds (EBA, FBA, and LOC) and target regions (dorsal and ventral) indicated a significant interaction between seed and target regions in terms of resting-state connectivity strength [*F*(2,60) = 13.03, *p* < 0.001], while the three-way interaction including hemisphere (left and right), seed (EBA, FBA, and LOC), and target region (dorsal and ventral) was not significant [*F*(2,60) = 2.65, *p* = 0.079]. Accordingly, the seed–target interaction was observed for both left hemisphere [*F*(2,60) = 7.92, *p* = 0.002] and right hemisphere [*F*(2,60) = 12.23, *p* < 0.001; all Bonferroni corrected].

The underlying 2 × 2 interaction with seed regions EBA and LOC was significant [*F*(1,30) = 14.68, *p* = 0.003]. The 2 × 2 interaction with seed regions EBA and FBA was also significant [*F*(1,30) = 25.87, *p* < 0.001]. The interaction between seed regions LOC and FBA and target regions (dorsal and ventral) was not significant [*F*(1,30) = 0.61, *p* > 0.10; all Bonferroni corrected]. The same patterns were observed for both hemispheres. As shown in Fig. 5, EBA shows relatively stronger connectivity to the dorsal target ROI compared to LOC and FBA, which both show relatively stronger connectivity with the ventral stream ROI. Whereas LOC and FBA show greater connectivity strength to ventral stream ROIs [LOC: *F*(1,30) = 5.03, *p* = 0.032; FBA: *F*(1,30) = 10.74, *p* = 0.003], the difference for EBA between dorsal and ventral target regions was only a trend towards stronger connectivity with the dorsal-stream ROI [*F*(1,30) = 3.24, *p* = 0.082].

A detailed analysis of the connectivity strength between the seed regions and all regions of the HCP-MMP1.0 atlas individually shows that these effects are consistent over a large number of regions and are not driven by any outliers (Supplementary material S3). This analysis shows that the findings presented in Fig. 5 are robust across the whole of the lateral parietal and inferior temporal cortex and do not depend on minor and arbitrary changes to our definition of dorsal and ventral stream regions of interest.

##### Seed-region classification

**Fig. 6:**
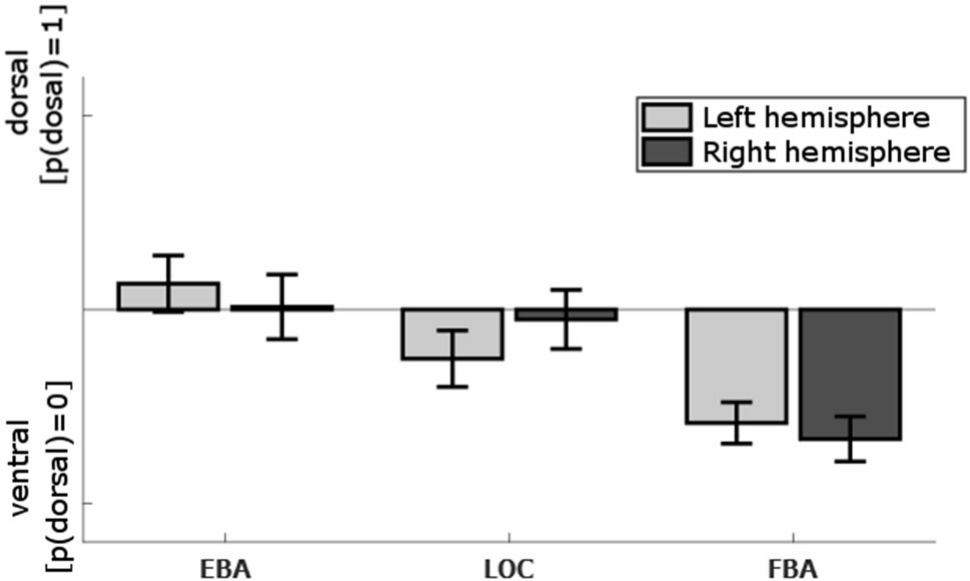
Classification result of seed regions EBA, LOC, and FBA classified as either ‘dorsal’ or ‘ventral’ based on whole-brain connectivity patterns of several dorsal and ventral stream areas (see Table 2; Definition of dorsal and ventral stream target regions) for left and right hemispheres. Seed regions differ in classification outcome: EBA is consistently classified more dorsal than FBA, with LOC between EBA and FBA. *Error bars* indicate the standard error of the mean. *Bars* represent the probability of a seed region being classified as ‘dorsal’, ranging from 0 (always ventral) to 1 (always dorsal). *Bars* are centred to *p* = 0.5, i.e., equally likely to be classified as dorsal or ventral. Presented classification outcomes are based on average classification outcomes over different classifier parameters (i.e., *k* in a *k*-nearest-neighbours classification). Full results for different *k* values are presented in the supplementary material (Figure S2)

Next, complementary to the analyses of connectivity strength, we aimed to classify whole-brain connectivity patterns of the seed regions (EBA, FBA, and LOC) as either ‘dorsal’ or ‘ventral’. We based the classification on the resemblance of the seeds’ whole-brain connectivity patterns to those of the segmented regions from the HCP-MMP1.0 atlas (Glasser et al. 2016) used to define dorsal and ventral streams (see Definition of dorsal and ventral stream target regions; Table 2). Figure 6 shows the average classification outcome for different classifiers (i.e., different numbers of neighbours *k* in a *k*-nearest-neighbour classification, see “Methods”). However, *k* was included as factor in the analyses. Classification outcome differed between seed regions for both hemispheres [left: *χ* ^2^(2,558) = 79.93, *p* < 0.001; right: *χ* ^2^(2,558) = 104.42, *p* < 0.001; Friedman’s non-parametric test], with a significant interaction between seed region and hemisphere [*F*(2,360) = 14.70, *p* < 0.001]. In the left hemisphere, EBA was more likely to be classified as dorsal compared to both LOC [*χ* ^2^(1,372) = 24.29, *p* < 0.001] and FBA [*χ* ^2^(1,372) = 79.09, *p* < 0.001], and LOC was more likely classified as dorsal than FBA [*χ* ^2^(1,372) = 23.73, *p* < 0.001]. In the right hemisphere, there was no difference in classification outcome between EBA and LOC (*p* > 0.10), but both EBA and LOC were more likely to be classified as dorsal than FBA [EBA vs. FBA: *χ* ^2^(1,372) = 100.83, *p* < 0.001; LOC vs. FBA: *χ* ^2^(1,372) = 77.29, *p* < 0.001]. Interestingly, classification outcome depended on the level of *k*, as was shown in a number of interactions with *k* [hemisphere × seed region × *k*: *F*(12,360) = 4.24, *p* < 0.001; seed region × *k*: *F*(12,360) = 12.36, *p* < 0.001; hemisphere × *k*: *F*(6,360) = 4.06, *p* = 0.001; and an effect of *k*: *F*(6,360) = 2.58, *p* = 0.020]. Visual inspection of the data revealed that with small *k* (*k* < 5), all seed regions are classified predominantly ventral or neutral, whereas classification outcome for EBA changes towards *dorsal* with larger *k* (4 < *k* < 9). Importantly, the relationship between the regions of interest is independent of *k*, with the dorsal classification of EBA > LOC > FBA in the left hemisphere and EBA ≅ LOC > FBA in the right hemisphere. Classification outcome for FBA is consistently *ventral*, irrespective of *k*. Detailed results are presented in the supplementary material (Figure S2).

##### Probabilistic tractography of diffusion-weighted MRI

**Fig. 7:**
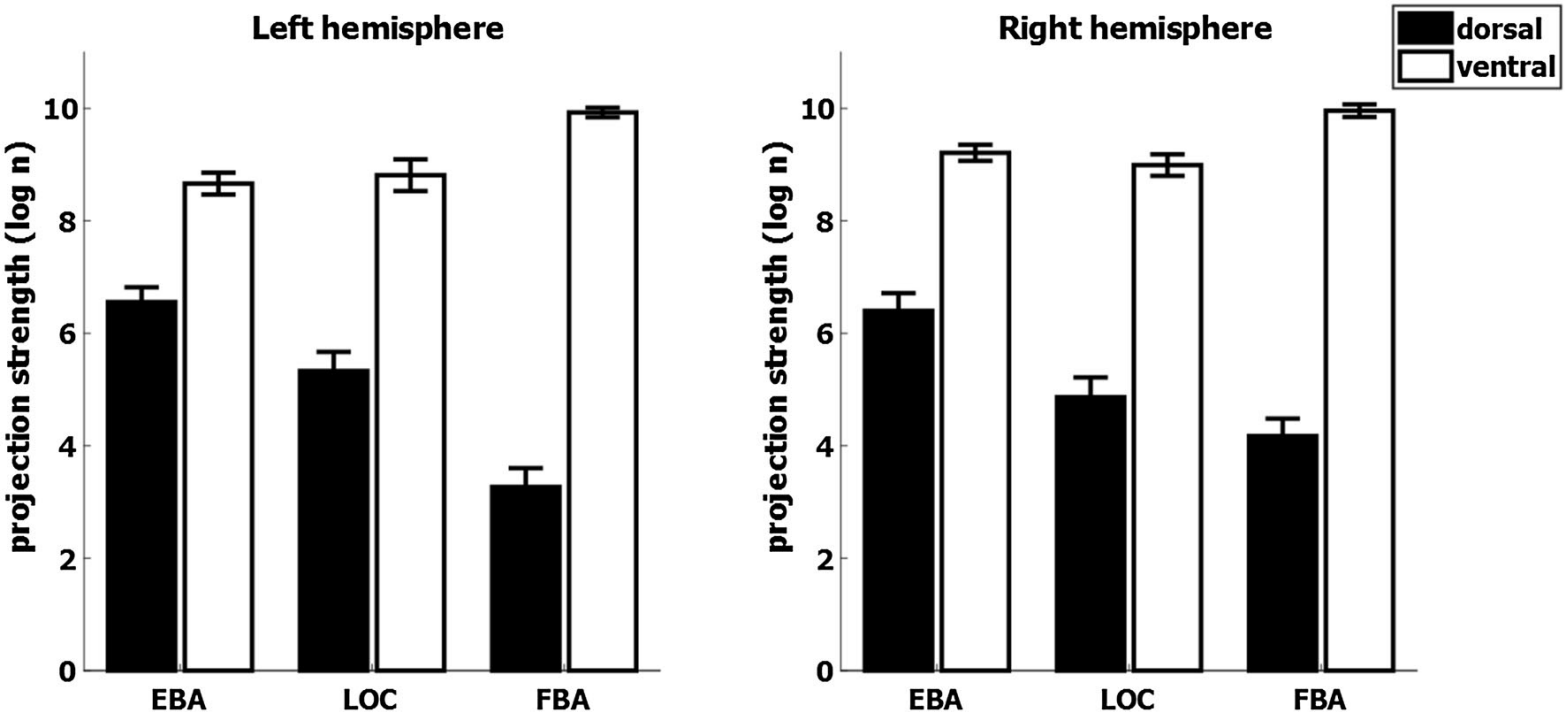
Connectivity strength of EBA, FBA, and LOC with ventral and dorsal regions indexed using probabilistic tractography of diffusion-weighted MRI. All three seed regions show more projections to ventral than dorsal regions, but the relative distribution differed: EBA is characterized by relatively stronger dorsal projections and FBA by relatively stronger ventral projections. *Error bars* indicate the standard error of the mean

Finally, we aimed to complement and confirm the resting-state fMRI approach using probabilistic tractography on diffusion-weighted MRI data. Tractography showed that all three seed regions had more projections to ventral than dorsal regions in absolute terms. This is expected given their spatial proximity to the ventral target. However, it also revealed that the relative distribution of ventral and dorsal projections differed per region: EBA was characterized by strong dorsal projections and FBA by strong ventral projections, with an intermediate pattern for LOC (Fig. 7). Specifically, we found significant interactions of seed by target region in connection strength (log-transformed tract probability) in both hemispheres [left: *F*(2,62) = 55.32, *p* < 0.001; right: *F*(2,62) = 21.61, *p* < 0.001]. This index differed between hemispheres, as indicated by the significant three-way interaction between seed region, target region, and hemisphere [*F*(2,62) = 4.00, *p* = 0.023], likely caused by hemispheric differences of FBA as shown by a significant interaction with factors hemisphere and target region for FBA [*F*(1,31) = 6.65, *p* = 0.015], which was not significant for EBA and LOC (both *p* > 0.10). Post-hoc tests revealed that seed regions differed significantly in terms of their probability of connecting to the dorsal-stream ROIs in both hemispheres [left: *F*(2,62) = 36.82, *p* < 0.001; right: *F*(2,62) = 16.81, *p* < 0.001]. Specifically, EBA’s probability of connecting to the dorsal stream was stronger than LOC’s and FBA’s [left: *t*(31) = 3.42, *p* = 0.006; *t*(31) = 8.98, *p* < 0.001; right: *t*(31) = 6.58, *p* < 0.001; *t*(31) = 5.06, *p* < 0.001]. There was a significant difference between LOC and FBA in the left hemisphere, but not in the right hemisphere [left: *t*(31) = 4.76, *p* < 0.001; right: *t*(31) = 1.49, *p* > 0.10; all Bonferroni corrected]. Similarly, seed regions differed significantly in terms of their probability of connecting to the ventral stream ROIs in both hemispheres [left: *F*(2,62) = 14.67, *p* < 0.001; right: *F*(2,62) = 12.80, *p* < 0.001]. Specifically, in both hemispheres, FBA had a stronger probability of connecting to ventral stream than EBA and LOC [left: *t*(31) = 6.17, *p* < 0.001; *t*(31) = 4.06, *p* = 0.001; right: *t*(31) = 4.45, *p* < 0.001; *t*(31) = 4.58, *p* < 0.001]. EBA and LOC did not differ significantly in either hemisphere (all *p* > 0.10, all Bonferroni corrected). Although FBA is closer in distance to ventral stream regions than EBA and LOC, these effects cannot simply be explained by a bias of distance between the seed and the target regions: all seed regions are similarly distant from dorsal-stream regions and EBA and LOC are equidistant from inferior temporal, yet a striking difference in connectivity probability was observed. The probabilistic tractography analysis confirms the rs-fMRI finding, indicating that EBA is relatively strongly connected with the dorsal stream.

A detailed analysis of the connection strength between the seed regions and all regions of the HCP-MMP1.0 atlas individually shows that these effects are consistent over a large number of regions and are not driven by any outliers (Supplementary material S4), showing that the findings presented in Fig. 7 are robust across the whole of the lateral parietal and inferior temporal cortex.

## Discussion

This study tests whether EBA has stronger connectivity with the dorsal visuomotor stream than two nearby perceptual areas in the occipito-temporal cortex, LOC and FBA. The main finding of this study is that EBA is more strongly connected to parietal regions than FBA and LOC, both functionally and structurally. This observation clarifies the ongoing debate on EBA function (Downing and Peelen 2011, 2016), providing anatomical evidence for the notion that EBA is more closely engaged with portions of the parietal cortex than other occipito-temporal areas. EBA’s connectivity profile supports the suggestion that its contributions to goal-oriented behaviour are different from those of other portions of the ventral visual stream (Kühn et al. 2011; van Nuenen et al. 2012; Zimmermann et al. 2012, 2016).

### Dorsal-stream affinity of the extrastriate body area

The dw-MRI tractography index of structural connectivity reflected the proximity and connectivity of all investigated occipito-temporal regions to the ventral stream. In addition, it revealed that EBA’s affinity with the dorsal stream is markedly stronger than that of LOC and FBA (Fig. 7), revealing a more extensive connectivity profile than previously identified (Beer et al. 2013). This effect was present for both the left and right hemispheric homologues. The resting-state functional coupling of EBA also revealed a greater affinity, both in strength and profile, with the dorsal stream than with the ventral stream (Fig. 5). At a whole-brain level, EBA has strong functional connections with occipital regions. In addition, EBA is functionally coupled with the superior parietal lobe, the parietal operculum, and the postcentral gyrus (Fig. 2), in line with previous studies showing that EBA is connected to regions involved in various action related processes (e.g., Beer et al. 2013; Zimmermann et al. 2013; Orgs et al. 2016; Simos et al. 2017). These parietal regions are involved in somatosensory processing (Dijkerman and de Haan 2007), in the integration of somatosensory and visual information during reaching/grasping movements (Fogassi and Luppino 2005), and in the estimation of future body states during action execution (Wolpert et al. 1998). Those parietal regions could provide EBA with access to somatosensory representations of one’s own current body posture. This functional connectivity profile fits with the observation that EBA is sensitive to discrepancies between one’s own current body posture and predicted body postures (Arzy et al. 2006; Zimmermann et al. 2012, 2013; Limanowski et al. 2014; Limanowski and Blankenburg 2016). It remains unclear whether the whole EBA or only a portion has access to both visual and somatosensory information, similar to how the lateral occipital complex contains a sub-section sensitive to both visual and tactile object information (Amedi et al. 2001). Future investigations could test whether EBA could be further subdivided in modality-related integrative units, as other parts of the lateral occipito-temporal cortex (see Bracci et al. 2012).

The exceptionally strong connectivity of EBA with postcentral, opercular, and dorsomedial portions of the parietal cortex does not fit with a purely visuo-perceptual role for this area. Access to somatosensory information would be irrelevant for an EBA devoted to identify visually presented body stimuli, or to process the perceptual consequences of executed motor acts (Downing et al. 2001; Downing and Peelen 2011). In contrast, knowing the current postural configuration of one’s own body is crucial for an EBA involved in motor control (van Nuenen et al. 2012; Zimmermann et al. 2012, 2016). EBA’s access to both visual and haptic information (Kitada et al. 2009, 2014) supports the notion that this region biases the sensorimotor transformations implemented in the parieto-frontal circuits with a postural goal-state derived from learned knowledge (Verhagen et al. 2012).

### EBA and FBA

FBA, which is consistently co-activated with EBA (Peelen and Downing 2005; Weiner and Grill-Spector 2010), shows weaker connectivity than EBA with dorsal-stream regions. This could suggest that FBA plays primarily a perceptual role, namely, the role originally proposed for EBA (Downing et al. 2001). Accordingly, FBA and EBA have been proposed to be hierarchically organized regions involved in body perception with FBA representing more holistic, whole-body information than EBA, as well as dynamic movements and identity (Hodzic et al. 2009; Ewbank et al. 2011; Orgs et al. 2016). Alternatively, the FBA connectivity isolated in this study raises the possibility that this region is also involved in providing desired goal states to action plans, although through a different circuit than EBA. Namely, FBA connectivity with areas 45, 46, and IFS could mediate perceptual influences on motor planning through prefrontal cortex, such as the selection of action targets and objects according to abstract goals (Milner and Goodale 2008). These ventral visual stream influences on action selection would become relevant only during late planning stages of actions (Milner and Goodale 2008), in contrast to the direct and early influences on motor behaviour exerted by EBA (Zimmermann et al. 2016).

### EBA and LOC

LOC is consistently associated with object perception and recognition (Malach et al. 1995; Amedi et al. 2001; Grill-Spector et al. 2001; Kourtzi and Kanwisher 2001), alike EBA’s involvement in body perception. The similarities extend further, as not only EBA, but also LOC has been associated with action planning (Verhagen et al. 2008; Gallivan et al. 2015). Specifically, LOC is thought to provide perceptual information about objects used for grasping (Verhagen et al. 2008). EBA, in contrast, is thought to represent desired body postures used during planning and execution of goal-directed actions (Zimmermann et al. 2012, 2016). Previously, we have suggested LOC contributes to the initiation of action planning by providing object-based priors (Verhagen et al. 2012). Recently, we have shown that EBA critically shapes the earliest stages of action planning when one’s body posture is relevant for the action outcome (Zimmermann et al. 2016). This highlights the possibility that EBA might provide the dorsal stream with perceptual-based action priors, similar to LOC. Importantly, the functional contributions of LOC and EBA to action and perception are reflected in their respective connectivity profiles, highlighting access to both ventral- and dorsal-stream areas (Figs. 2, 4). Connections of EBA with dorsal-stream regions are stronger than those of LOC, putatively reflecting a more direct involvement in motor control for EBA (Figs. 4, 5, 7). Detailed analyses of their connectivity profiles may reveal specific connections along the dorsal stream that correspond to the dissociable contributions these regions may have to action.

### EBA and MT+

The cortical extent of EBA has been suggested to overlap at least partly with motion area MT+ (Orban et al. 2004; Peelen et al. 2006; Downing et al. 2007; Ferri et al. 2013). It is yet not clear whether this region’s sensitivity to static bodies and moving dots, two very different stimuli, is driven by the same or different neuronal populations. The current study was not focused on resolving this issue or on making inferences specific to the portion of EBA that does not overlap with hMT+. Rather, here, we test a long-standing assumption on EBA functionality, namely, that EBA is a canonical category-specific perceptual area with connections correspondingly biased towards the ventral visual stream. The current findings do not support this assumption. Instead, the observed pattern of connectivity opens the possibility that EBA provides an interface between action and perception, similar to the role of MT+ in planning and control of goal-directed actions (Maunsell and van Essen 1983; Ungerleider and Desimone 1986; Lewis and Van Essen 2000).

### Interpretational issues

Resting-state connectivity and diffusion MRI do not provide information about the directionality of connections. The suggestion that brain regions in the occipito-temporal cortex project onto the motor system implies a directed information flow from occipito-temporal to dorsal-stream regions. Similarly, accounts that emphasize how motor information is used to predict sensory consequences of actions would require the same connections, in the opposite direction. Findings from a recent transcranial magnetic stimulation study, on the same participants, complement the current observations with causal information about the direction and the temporal relevance of these connections, showing that EBA influences action planning well before IPS (Zimmermann et al. 2016).

Resting-state connectivity is an indirect measure of anatomy that cannot distinguish between direct and indirect connections (Honey et al. 2009; Passingham 2013). For instance, correlation of BOLD responses between two regions can be caused by a third region that projects to both, in the absence of a direct connection between the two BOLD-correlated regions. Nonetheless, this approach has proven to be very sensitive to differences in correlation patterns between regions, also in the occipito-temporal cortex (Hutchison et al. 2014). Diffusion MRI tractography has different limitations; for instance, its results are strongly biased by the distance between seed- and target regions. Moreover, diffusion tractography methods have trouble distinguishing between crossing and curved fibres within a voxel, leading to a sub-optimal balance between sensitivity and specificity (Thomas et al. 2014). Although diffusion tractography has been repeatedly proven to closely match the golden-standard of tracing results (Croxson et al. 2005; Dauguet et al. 2007; Jbabdi et al. 2013; Azadbakht et al. 2015), the goal of dw-MRI tractography in this study is dissimilar from that of tracing studies. We aim to quantify the probability of regions being part of the same connectional system, and markedly do not aim to qualify the presence of single-synapse connections between these regions. Furthermore, the current analyses are designed to complement rs-fMRI and dw-MRI strengths while minimizing their weaknesses. For instance, we have used subject-specific localised seed regions and a priori target regions, to balance specificity and sensitivity. Moreover, in line with most diffusion tractography studies, we do not draw inferences on absolute connectivity probabilities, but compare relative values across regions with similar distances.

Some analyses resulted in hemispheric differences, suggesting that left and right hemispheric regions may have different specializations, consistent with suggestions of hemispheric specialization in motor control and perception (Schluter et al. 1998; Downing et al. 2001; de Lange et al. 2005; Peelen and Downing 2005; Arzy et al. 2006; Willems et al. 2009; Zimmermann et al. 2012; Limanowski et al. 2014). However, in this study, no clear patterns were observed with respect to hemispheric differences and findings of hemispheric differences were not consistent over complementary analyses. Future studies are required to investigate whether and how structural and functional connectivity of the investigated regions differ between hemispheres.

In this study we focus on the motor aspects of the dorsal stream, but it is worth noting that this circuit is also involved in visuospatial perception (e.g., Ungerleider and Haxby 1994). It is conceivable that differences in dorsal-stream connectivity of EBA, LOC, and FBA reflect differences in the regions’ contributions to visuospatial perception. Namely, one could speculate that recognition of body parts could contribute both to the estimation of the relative position of other persons and their posture to oneself (putatively mediated by EBA) and to the identification of the owner of the body part (putatively mediated by FBA based on holistic body representations; Taylor et al. 2007). In this framework, LOC would be expected to show an intermediate connectivity profile, given that relative position and identity are often both highly relevant for objects. Along these lines, regions computing identification per se, such as FFA processing face information, are, therefore, expected to have a more ventral connectivity profile. Conversely, this framework also predicts that the parahippocampal place area (PPA) has a comparably dorsally oriented connectivity profile, as the relative spatial location of places is often relevant in addition to the identification of these places.

## Conclusions

Here, we provide anatomical evidence that the extrastriate body area has strong interactions with parietal cortex, allowing it to exchange information with dorsal-stream areas. Diffusion tractography confirmed the relative dorsal-stream affinity of EBA. Contrasting with EBA, the fusiform body area could be robustly classified as a ventral region based on its connectivity profile. It is characterized by strong connections to higher perceptual areas in the inferior temporal cortex and a lack of marked connections to posterior parietal and postcentral regions involved in motor control. The lateral occipital complex revealed an intermediate pattern with varying affinity for ventral and dorsal visual streams, suggesting that it might not only serve as a gateway for ventral, but also for dorsal-stream processing. These observations provide an anatomical ground for the suggestion that EBA is not only involved in body perception (Downing et al. 2001; Downing and Peelen 2011), but also contributes to action planning by anticipating body states (Zimmermann et al. 2012, 2016).

Taken together, this study adds to a growing body of the literature suggesting that the boundary between dorsal and ventral visual processing streams is not as clear as it was suggested initially (Goodale and Milner 1992). In fact, studies reporting existence of several parallel processing streams (Kravitz et al. 2011, 2013) and observations that regions are well connected to both streams, such as those reported here (see also Orgs et al. 2016), challenge this initial view (see also Milner 2017).

## Electronic supplementary material

Below is the link to the electronic supplementary material.

Supplementary material 1 (DOCX 8450 kb)
