# Supplementary material for: Is the extrastriate body area part of the dorsal visuomotor stream?
Source: Brain Struct Funct. 2017 Jul 12;223(1):31–46. doi: 10.1007/s00429-017-1469-0 (PMC5772142; doi:10.1007/s00429-017-1469-0)
Supplement: Supplementary file 1 — Supplementary material 1 (DOCX 8450 kb) [file 429_2017_1469_MOESM1_ESM.docx]

***Supplementary material***

Article title: Is the extrastriate body area part of the dorsal visuomotor stream?

Journal: Brain Structure and Function

Authors: Marius Zimmermann*^1,2^, Rogier B. Mars^1,3^, Floris P. de Lange^1^, Ivan Toni^1^, Lennart Verhagen^4^

*^1^ Donders Institute for Brain, Cognition and Behaviour, Radboud University Nijmegen, The Netherlands
^2^ Department of Psychology, Stockholm University, Stockholm, Sweden
^3^ Wellcome Centre for Integrative Neuroimaging, Centre for Functional MRI of the Brain (FMRIB), Nuffield Department of Clinical Neurosciences, John Radcliffe Hospital, University of Oxford, Oxford, UK
^4^ Department of Experimental Psychology, University of Oxford, Oxford, UK*

* corresponding author

Marius Zimmermann
email: [marius.zimmermann@psychology.su.se](mailto:marius.zimmermann@psychology.su.se)
phone: +46 8 163805
fax: +46 8 159342

**Table S1.** List of regions covered by clusters of comparisons between seed-regions, limited to one local maximum per region. Only significant clusters are considered (p(FWE)<.05), based on voxel-wise, uncorrected threshold of p<.001 (as Figure 4). Labelling is done according to the anatomy toolbox (Eickhoff et al. 2005).

**[LH] EBA > FBA**

| **Region** | **X** | **Y** | **Z** | **T-value** |
| --- | --- | --- | --- | --- |
| L Middle Occipital Gyrus | -44 | -78 | 10 | 11.73 |
| R Middle Temporal Gyrus | 50 | -74 | 10 | 10.37 |
| L Cuneus | -4 | -86 | 26 | 10.05 |
| R Cuneus | 6 | -84 | 26 | 9.22 |
| L Superior Occipital Gyrus | -18 | -88 | 30 | 8.95 |
| R Superior Occipital Gyrus | 24 | -86 | 32 | 8.46 |
| R Middle Occipital Gyrus | 30 | -88 | 22 | 8.29 |
| L Calcarine Gyrus | -14 | -68 | 10 | 7.88 |
| L Superior Temporal Gyrus | -42 | -32 | 14 | 7.36 |
| L Heschls Gyrus | -36 | -24 | 12 | 7.12 |
| R Insula Lobe | 42 | -8 | 12 | 6.45 |
| L Rolandic Operculum | -46 | -6 | 6 | 6.42 |
| R Superior Temporal Gyrus | 66 | -38 | 16 | 6.21 |
| R Heschls Gyrus | 42 | -26 | 14 | 6.20 |
| R Lingual Gyrus | 12 | -68 | 0 | 6.13 |
| L Lingual Gyrus | -22 | -58 | -2 | 6.07 |
| R Superior Parietal Lobule | 26 | -48 | 58 | 5.58 |
| R Rolandic Operculum | 56 | -4 | 10 | 5.50 |
| L Postcentral Gyrus | -26 | -44 | 56 | 5.31 |
| L Superior Parietal Lobule | -32 | -44 | 58 | 5.13 |
| R Inferior Occipital Gyrus | 44 | -66 | -14 | 5.08 |
| R Fusiform Gyrus | 28 | -74 | -14 | 4.83 |
| R Postcentral Gyrus | 56 | -6 | 36 | 4.81 |
| R Precentral Gyrus | 54 | -14 | 42 | 4.75 |
| L Precentral Gyrus | -36 | -28 | 58 | 4.66 |
| L Insula Lobe | -36 | 6 | 4 | 4.38 |
| R SupraMarginal Gyrus | 48 | -32 | 24 | 4.19 |
| L Precuneus | -4 | -48 | 62 | 3.80 |
| R Precuneus | 14 | -60 | 60 | 3.79 |
| L Paracentral Lobule | -12 | -32 | 70 | 3.77 |
| L Posterior-Medial Frontal | -8 | -10 | 70 | 3.69 |
| L Middle Temporal Gyrus | -68 | -12 | 0 | 3.62 |
| R Superior Frontal Gyrus | 28 | -12 | 64 | 3.28 |
| L ACC | -2 | 36 | 16 | 4.3 |

**[LH] EBA > LOC**

| **Region** | **X** | **Y** | **Z** | **T-value** |
| --- | --- | --- | --- | --- |
| L Superior Temporal Gyrus | -52 | -32 | 16 | 7.44 |
| L Calcarine Gyrus | -22 | -56 | 8 | 6.46 |
| L Rolandic Operculum | -48 | -10 | 12 | 6.39 |
| L Middle Occipital Gyrus | -44 | -82 | 26 | 5.26 |
| L Heschls Gyrus | -34 | -24 | 14 | 5.16 |
| R Calcarine Gyrus | 8 | -62 | 18 | 5.09 |
| L Insula Lobe | -34 | -20 | 12 | 5.05 |
| L Angular Gyrus | -54 | -66 | 36 | 4.73 |
| L Postcentral Gyrus | -52 | -14 | 24 | 3.97 |
| R Precuneus | 14 | -52 | 18 | 3.27 |
| R Rolandic Operculum | 52 | -12 | 14 | 6.33 |
| R Middle Temporal Gyrus | 48 | -66 | 12 | 6.31 |
| R Superior Temporal Gyrus | 68 | -40 | 16 | 6.14 |
| R SupraMarginal Gyrus | 46 | -32 | 24 | 5.75 |
| R Postcentral Gyrus | 64 | -8 | 36 | 4.86 |
| R Middle Occipital Gyrus | 44 | -80 | 32 | 4.14 |
| R Precentral Gyrus | 62 | 4 | 32 | 4.07 |
| R Angular Gyrus | 48 | -76 | 34 | 4.05 |
| L Superior Medial Gyrus | 0 | 48 | 38 | 5.24 |
| L Superior Frontal Gyrus | -18 | 44 | 40 | 3.69 |
| L Middle Frontal Gyrus | -20 | 48 | 30 | 3.43 |
| R Superior Medial Gyrus | 6 | 64 | 26 | 3.39 |
| L Precentral Gyrus | -62 | 6 | 24 | 5.45 |
| L SupraMarginal Gyrus | -62 | -20 | 42 | 4.18 |
| L Inferior Parietal Lobule | -54 | -22 | 44 | 3.63 |
| R MCC | 2 | 8 | 32 | 3.94 |
| R ACC | 4 | 16 | 26 | 3.79 |

**[LH] FBA > EBA**

| **Region** | **X** | **Y** | **Z** | **T-value** |
| --- | --- | --- | --- | --- |
| L Inferior Temporal Gyrus | -44 | -46 | -18 | 12.12 |
| R ParaHippocampal Gyrus | 18 | -26 | -14 | 8.87 |
| L Fusiform Gyrus | -34 | -38 | -18 | 8.62 |
| L ParaHippocampal Gyrus | -16 | -22 | -18 | 8.30 |
| R Superior Temporal Gyrus | 44 | -2 | -14 | 6.52 |
| L Middle Temporal Gyrus | -46 | 0 | -16 | 6.36 |
| L Superior Temporal Gyrus | -44 | -2 | -14 | 6.32 |
| L Olfactory cortex | -22 | 4 | -18 | 6.28 |
| R Middle Orbital Gyrus | 24 | 32 | -22 | 5.79 |
| R Amygdala | 18 | 0 | -16 | 5.54 |
| L Temporal Pole | -48 | 20 | -14 | 5.53 |
| L Cerebellum (III) | -10 | -38 | -22 | 5.49 |
| R Rectal Gyrus | 10 | 22 | -16 | 5.28 |
| R IFG (p. Orbitalis) | 50 | 48 | -12 | 5.24 |
| L IFG (p. Triangularis) | -48 | 42 | 0 | 5.12 |
| R Mid Orbital Gyrus | 4 | 22 | -10 | 5.01 |
| L Cerebellum (Crus 1) | -32 | -62 | -38 | 4.82 |
| L IFG (p. Orbitalis) | -40 | 28 | -16 | 4.71 |
| L Cerebellum (Crus 2) | -46 | -76 | -40 | 4.37 |
| L Mid Orbital Gyrus | -2 | 28 | -12 | 4.37 |
| L Middle Orbital Gyrus | -20 | 62 | -12 | 4.20 |
| L Superior Orbital Gyrus | -14 | 56 | -10 | 4.18 |
| L Thalamus | -4 | -10 | 4 | 4.12 |
| L Rectal Gyrus | -10 | 34 | -22 | 4.05 |
| L Cerebellum (VII) | -38 | -62 | -52 | 4.05 |
| L Hippocampus | -30 | -14 | -12 | 3.92 |
| L Cerebellum (VIII) | -38 | -58 | -56 | 3.56 |
| L Posterior-Medial Frontal | -6 | 20 | 52 | 7.13 |
| R Posterior-Medial Frontal | 2 | 14 | 58 | 5.73 |
| L Superior Frontal Gyrus | -14 | 16 | 48 | 4.93 |
| L Middle Frontal Gyrus | -28 | 14 | 44 | 4.71 |
| L Superior Medial Gyrus | -4 | 30 | 46 | 4.69 |
| R Superior Medial Gyrus | 6 | 36 | 42 | 3.41 |
| R Cerebellum (Crus 1) | 32 | -66 | -32 | 5.79 |
| R Cerebellum (VI) | 24 | -66 | -32 | 5.58 |
| R Cerebellum (Crus 2) | 30 | -84 | -46 | 4.72 |
| R Cerebellum (VII) | 26 | -78 | -50 | 4.41 |
| R Inferior Temporal Gyrus | 54 | -38 | -18 | 6.70 |
| R Middle Temporal Gyrus | 58 | -20 | -16 | 4.39 |
| L Inferior Parietal Lobule | -52 | -54 | 50 | 5.00 |
| R Angular Gyrus | 48 | -60 | 54 | 5.36 |
| R Inferior Parietal Lobule | 50 | -52 | 56 | 4.72 |
| R Superior Orbital Gyrus | 18 | 56 | -10 | 4.38 |

**[LH] FBA > LOC**

| **Region** | **X** | **Y** | **Z** | **T-value** |
| --- | --- | --- | --- | --- |
| L Inferior Temporal Gyrus | -44 | -46 | -18 | 13.00 |
| L Fusiform Gyrus | -34 | -38 | -18 | 8.59 |
| L Hippocampus | -16 | -20 | -18 | 7.18 |
| L Amygdala | -20 | 0 | -14 | 5.44 |
| L Pallidum | -12 | 4 | -6 | 4.04 |
| L Cerebellum (III) | -10 | -38 | -22 | 3.70 |
| L Posterior-Medial Frontal | -6 | 20 | 50 | 5.44 |
| L Middle Frontal Gyrus | -24 | 14 | 54 | 5.28 |
| L Superior Medial Gyrus | 0 | 44 | 36 | 4.87 |
| L Precentral Gyrus | -44 | 10 | 34 | 4.78 |
| L IFG (p. Triangularis) | -54 | 18 | 22 | 4.74 |
| L Superior Frontal Gyrus | -18 | 14 | 46 | 3.77 |
| R Superior Medial Gyrus | 2 | 30 | 46 | 3.70 |
| L Inferior Parietal Lobule | -54 | -56 | 48 | 5.58 |
| L Angular Gyrus | -42 | -74 | 42 | 4.51 |
| L Middle Occipital Gyrus | -38 | -82 | 38 | 4.46 |
| R Hippocampus | 14 | -4 | -16 | 4.61 |
| R ParaHippocampal Gyrus | 38 | -32 | -14 | 3.72 |
| R Olfactory cortex | 22 | 10 | -18 | 3.65 |
| R Cerebellum (VII) | 32 | -74 | -54 | 4.45 |
| R Cerebellum (Crus 2) | 36 | -86 | -38 | 3.90 |
| R Cerebellum (VI) | 26 | -66 | -30 | 5.01 |
| R Cerebellum (Crus 1) | 18 | -76 | -30 | 4.82 |
| R Caudate Nucleus | 18 | -12 | 22 | 3.45 |
| L Superior Temporal Gyrus | -44 | -2 | -14 | 4.40 |
| L IFG (p. Orbitalis) | -34 | 38 | -12 | 4.57 |
| R Rectal Gyrus | 6 | 32 | -18 | 4.33 |
| R Mid Orbital Gyrus | 8 | 24 | -14 | 3.78 |
| L Rectal Gyrus | 0 | 36 | -18 | 3.65 |
| R Inferior Temporal Gyrus | 52 | -40 | -18 | 4.26 |
| R Middle Temporal Gyrus | 60 | -36 | -14 | 4.20 |
| L Middle Orbital Gyrus | -48 | 48 | -4 | 3.65 |

**[LH] LOC > EBA**

| **Region** | **X** | **Y** | **Z** | **T-value** |
| --- | --- | --- | --- | --- |
| L Inferior Occipital Gyrus | -42 | -78 | -8 | 12.14 |
| L Fusiform Gyrus | -38 | -80 | -14 | 8.81 |
| L Calcarine Gyrus | -4 | -100 | -6 | 7.31 |
| R Inferior Occipital Gyrus | 40 | -86 | -12 | 7.12 |
| R Middle Occipital Gyrus | 34 | -82 | 2 | 5.72 |
| Cerebellar Vermis (6) | 0 | -72 | -6 | 5.16 |
| L Cerebellum (Crus 2) | -6 | -90 | -26 | 3.44 |
| R Posterior-Medial Frontal | 4 | 16 | 60 | 5.73 |
| L Posterior-Medial Frontal | -6 | 10 | 56 | 4.48 |
| L Cerebellum (IV-V) | -6 | -44 | -10 | 5.04 |
| Cerebellar Vermis (4/5) | 0 | -48 | -2 | 4.41 |
| R IFG (p. Orbitalis) | 38 | 24 | -12 | 4.59 |
| R Middle Orbital Gyrus | 28 | 42 | -18 | 5.11 |
| R Superior Orbital Gyrus | 16 | 46 | -20 | 3.65 |
| R Rectal Gyrus | 16 | 18 | -14 | 5.38 |

**[LH] LOC > FBA**

| **Region** | **X** | **Y** | **Z** | **T-value** |
| --- | --- | --- | --- | --- |
| L Inferior Occipital Gyrus | -42 | -78 | -6 | 14.54 |
| R Middle Occipital Gyrus | 36 | -80 | 0 | 10.91 |
| L Middle Occipital Gyrus | -30 | -98 | -2 | 10.34 |
| R Inferior Occipital Gyrus | 40 | -82 | -6 | 10.13 |
| R Calcarine Gyrus | 10 | -86 | 12 | 9.03 |
| R Cuneus | 10 | -88 | 16 | 9.00 |
| L Cuneus | -6 | -86 | 26 | 8.83 |
| R Superior Occipital Gyrus | 26 | -82 | 30 | 8.06 |
| R Fusiform Gyrus | 30 | -76 | -14 | 7.74 |
| L Calcarine Gyrus | -4 | -92 | 6 | 7.67 |
| L Lingual Gyrus | -6 | -74 | 0 | 7.02 |
| R Lingual Gyrus | 20 | -74 | -10 | 7.00 |
| L Cerebellum (IV-V) | -8 | -40 | -8 | 4.60 |
| L Rolandic Operculum | -48 | -2 | 2 | 5.12 |
| L Heschls Gyrus | -38 | -22 | 6 | 4.03 |
| L Superior Temporal Gyrus | -60 | -18 | 2 | 3.75 |
| R Superior Parietal Lobule | 28 | -56 | 58 | 4.67 |
| R Inferior Parietal Lobule | 30 | -48 | 54 | 4.17 |
| R Insula Lobe | 44 | -8 | 4 | 4.28 |
| R Rolandic Operculum | 52 | -2 | 4 | 3.86 |
| R Superior Temporal Gyrus | 56 | -2 | 4 | 3.86 |

**[RH] EBA > FBA**

| **Region** | **X** | **Y** | **Z** | **T-value** |
| --- | --- | --- | --- | --- |
| R Middle Temporal Gyrus | 48 | -72 | 10 | 15.32 |
| R Middle Occipital Gyrus | 46 | -78 | 10 | 13.58 |
| R Calcarine Gyrus | 8 | -66 | 8 | 8.54 |
| L Superior Occipital Gyrus | -22 | -86 | 24 | 8.37 |
| R Cuneus | 6 | -78 | 32 | 7.92 |
| L Calcarine Gyrus | 2 | -86 | 10 | 7.89 |
| L Cuneus | 0 | -86 | 16 | 7.81 |
| L Middle Occipital Gyrus | -40 | -84 | 10 | 7.75 |
| L Lingual Gyrus | -14 | -78 | 2 | 6.56 |
| R Superior Parietal Lobule | 20 | -60 | 62 | 5.95 |
| R Lingual Gyrus | 26 | -60 | 2 | 5.91 |
| R Precuneus | 10 | -50 | 62 | 4.43 |
| R Postcentral Gyrus | 18 | -44 | 70 | 4.12 |
| R Inferior Occipital Gyrus | 36 | -96 | -4 | 4.05 |
| L Fusiform Gyrus | -24 | -76 | -16 | 4.04 |
| L Superior Parietal Lobule | -18 | -70 | 46 | 3.28 |
| L Insula Lobe | -38 | -14 | 10 | 6.34 |
| L Heschls Gyrus | -40 | -26 | 12 | 5.68 |
| L Superior Temporal Gyrus | -50 | -36 | 16 | 4.81 |
| L IFG (p. Opercularis) | -46 | 8 | 8 | 3.36 |
| L Postcentral Gyrus | -66 | -20 | 18 | 3.33 |
| L Inferior Parietal Lobule | -24 | -54 | 52 | 5.04 |
| L Precuneus | -6 | -50 | 62 | 3.84 |
| R Heschls Gyrus | 46 | -22 | 10 | 7.15 |
| L ACC | 2 | 22 | 24 | 6.07 |
| R Superior Temporal Gyrus | 70 | -40 | 12 | 4.44 |

**[RH] EBA > LOC**

| **Region** | **X** | **Y** | **Z** | **T-value** |
| --- | --- | --- | --- | --- |
| R Middle Temporal Gyrus | 52 | -74 | 10 | 14.43 |
| R Middle Occipital Gyrus | 46 | -78 | 10 | 11.53 |
| L Middle Occipital Gyrus | -42 | -80 | 20 | 7.52 |
| L Calcarine Gyrus | -16 | -66 | 12 | 6.41 |
| R Calcarine Gyrus | 18 | -62 | 12 | 5.85 |
| R Superior Temporal Gyrus | 42 | -32 | 14 | 5.74 |
| R Cuneus | 8 | -74 | 32 | 5.63 |
| R Heschls Gyrus | 40 | -28 | 14 | 5.61 |
| L Superior Parietal Lobule | -18 | -58 | 60 | 5.26 |
| L Middle Temporal Gyrus | -52 | -64 | 2 | 5.02 |
| R Rolandic Operculum | 56 | -18 | 16 | 4.74 |
| R SupraMarginal Gyrus | 50 | -34 | 28 | 4.58 |
| R Superior Occipital Gyrus | 24 | -90 | 32 | 4.28 |
| R Insula Lobe | 42 | -8 | 14 | 3.83 |
| L Cuneus | -8 | -74 | 30 | 3.80 |
| L Superior Occipital Gyrus | -26 | -90 | 34 | 3.60 |
| L Precuneus | -12 | -72 | 34 | 3.57 |
| R Postcentral Gyrus | 66 | -14 | 30 | 3.24 |
| L Superior Temporal Gyrus | -48 | -36 | 22 | 6.16 |
| L Precentral Gyrus | -54 | 4 | 20 | 5.33 |
| L Insula Lobe | -36 | -12 | 12 | 5.11 |
| L SupraMarginal Gyrus | -64 | -32 | 24 | 4.16 |
| R Precuneus | 12 | -48 | 46 | 4.85 |
| R Superior Parietal Lobule | 14 | -56 | 62 | 4.83 |
| L ACC | 0 | 18 | 26 | 4.17 |

**[RH] FBA > EBA**

| **Region** | **X** | **Y** | **Z** | **T-value** |
| --- | --- | --- | --- | --- |
| R Fusiform Gyrus | 40 | -46 | -20 | 10.12 |
| R Hippocampus | 24 | -16 | -14 | 6.64 |
| R ParaHippocampal Gyrus | 38 | -32 | -14 | 5.98 |
| L Amygdala | -24 | -4 | -12 | 5.56 |
| L Hippocampus | -16 | -6 | -16 | 4.92 |
| R Amygdala | 28 | 2 | -24 | 4.83 |
| L Rectal Gyrus | 0 | 48 | -16 | 4.61 |
| R Rectal Gyrus | 2 | 50 | -18 | 4.58 |
| L Mid Orbital Gyrus | 0 | 46 | -12 | 4.42 |
| L Olfactory cortex | -4 | 14 | -12 | 4.26 |
| L Inferior Temporal Gyrus | -48 | -44 | -14 | 4.22 |
| L ParaHippocampal Gyrus | -12 | 2 | -22 | 4.11 |
| R Caudate Nucleus | 12 | 8 | -12 | 4.09 |
| L Superior Orbital Gyrus | -16 | 26 | -14 | 4.06 |
| L Middle Temporal Gyrus | -58 | -40 | -14 | 4.05 |
| L Fusiform Gyrus | -32 | -8 | -30 | 3.82 |
| R Insula Lobe | 30 | 14 | -16 | 3.79 |
| R Olfactory cortex | 4 | 20 | -10 | 3.75 |
| L Temporal Pole | -22 | 6 | -22 | 3.58 |
| R Superior Orbital Gyrus | 8 | 52 | -24 | 3.36 |
| R Temporal Pole | 52 | 12 | -18 | 5.88 |
| R Middle Temporal Gyrus | 62 | -8 | -14 | 4.90 |
| R IFG (p. Orbitalis) | 42 | 32 | -12 | 4.15 |
| R Superior Temporal Gyrus | 44 | -6 | -14 | 4.08 |
| R Inferior Temporal Gyrus | 44 | -10 | -28 | 3.74 |
| R Medial Temporal Pole | 50 | 8 | -30 | 3.48 |
| R Superior Medial Gyrus | 4 | 28 | 52 | 4.40 |
| L Posterior-Medial Frontal | -4 | 20 | 54 | 4.32 |
| L Superior Medial Gyrus | -4 | 34 | 60 | 4.28 |
| L Superior Frontal Gyrus | -12 | 26 | 54 | 3.28 |
| L IFG (p. Orbitalis) | -40 | 42 | -12 | 4.56 |
| R Cerebellum (VII) | 36 | -66 | -48 | 4.32 |
| R Cerebellum (Crus 2) | 24 | -76 | -42 | 4.25 |
| R Cerebellum (Crus 1) | 24 | -68 | -38 | 3.96 |
| R Cerebellum (VIII) | 22 | -74 | -50 | 3.86 |
| L Superior Temporal Gyrus | -48 | -6 | -12 | 4.58 |
| L Middle Frontal Gyrus | -34 | 10 | 54 | 4.27 |
| L Precentral Gyrus | -46 | 10 | 48 | 3.78 |
| L Angular Gyrus | -40 | -72 | 48 | 4.53 |
| R Angular Gyrus | 40 | -70 | 50 | 4.60 |

**[RH] FBA > LOC**

| **Region** | **X** | **Y** | **Z** | **T-value** |
| --- | --- | --- | --- | --- |
| R Fusiform Gyrus | 44 | -46 | -18 | 10.96 |
| R ParaHippocampal Gyrus | 34 | -32 | -16 | 7.84 |
| L Amygdala | -26 | -4 | -16 | 6.15 |
| L Hippocampus | -18 | -20 | -16 | 5.82 |
| L Inferior Temporal Gyrus | -38 | 6 | -36 | 5.42 |
| L Fusiform Gyrus | -22 | -44 | -18 | 5.15 |
| R Hippocampus | 26 | -18 | -14 | 5.08 |
| L Medial Temporal Pole | -28 | 14 | -36 | 4.50 |
| L Caudate Nucleus | -6 | 8 | -10 | 4.41 |
| R Amygdala | 32 | 4 | -26 | 4.24 |
| L ParaHippocampal Gyrus | -16 | 2 | -18 | 4.22 |
| L Temporal Pole | -50 | 16 | -14 | 4.19 |
| R Temporal Pole | 50 | 12 | -20 | 4.14 |
| R Olfactory cortex | 28 | 12 | -14 | 3.62 |
| L Angular Gyrus | -42 | -76 | 40 | 5.35 |
| L Middle Occipital Gyrus | -36 | -84 | 38 | 4.99 |
| R Angular Gyrus | 46 | -76 | 38 | 5.76 |
| R Middle Occipital Gyrus | 42 | -84 | 30 | 3.90 |
| R IFG (p. Triangularis) | 54 | 32 | 22 | 4.82 |
| R IFG (p. Orbitalis) | 52 | 40 | -4 | 3.72 |
| R IFG (p. Opercularis) | 58 | 20 | 36 | 3.35 |
| L Rectal Gyrus | -10 | 30 | -14 | 3.99 |
| R Mid Orbital Gyrus | 4 | 42 | -14 | 3.91 |
| L Mid Orbital Gyrus | -2 | 50 | -10 | 3.81 |
| L Superior Medial Gyrus | -4 | 60 | 0 | 3.63 |
| R Rectal Gyrus | 4 | 46 | -18 | 3.57 |
| L Superior Orbital Gyrus | -18 | 30 | -14 | 3.28 |
| R Cerebellum (VIII) | 20 | -70 | -54 | 4.16 |
| R Cerebellum (VII) | 34 | -72 | -52 | 3.80 |
| R Cerebellum (Crus 2) | 24 | -78 | -46 | 3.68 |
| L Superior Temporal Gyrus | -50 | -6 | -12 | 5.39 |
| L Middle Temporal Gyrus | -64 | -6 | -14 | 3.55 |
| L Cerebellum (Crus 2) | -18 | -80 | -46 | 3.98 |
| L SupraMarginal Gyrus | -46 | -36 | 28 | 4.45 |
| R Superior Temporal Gyrus | 56 | -4 | -14 | 4.55 |

**[RH] LOC > EBA**

| **Region** | **X** | **Y** | **Z** | **T-value** |
| --- | --- | --- | --- | --- |
| R Inferior Occipital Gyrus | 42 | -78 | -8 | 10.02 |
| R Lingual Gyrus | 24 | -94 | -10 | 5.49 |
| L Fusiform Gyrus | -38 | -84 | -14 | 5.11 |
| L Calcarine Gyrus | -2 | -98 | -10 | 4.49 |
| L Inferior Occipital Gyrus | -28 | -94 | -8 | 4.40 |
| L Lingual Gyrus | -22 | -90 | -18 | 4.02 |
| L Posterior-Medial Frontal | -2 | 18 | 62 | 5.05 |
| R Posterior-Medial Frontal | 6 | 8 | 70 | 4.41 |
| L Superior Medial Gyrus | -2 | 30 | 62 | 3.66 |
| L Inferior Temporal Gyrus | -60 | -44 | -16 | 4.27 |

**[RH] LOC > FBA**

| **Region** | **X** | **Y** | **Z** | **T-value** |
| --- | --- | --- | --- | --- |
| R Inferior Occipital Gyrus | 46 | -76 | -8 | 12.50 |
| L Middle Occipital Gyrus | -32 | -92 | -4 | 9.08 |
| L Inferior Occipital Gyrus | -44 | -80 | -4 | 8.60 |
| L Fusiform Gyrus | -32 | -82 | -14 | 8.12 |
| R Calcarine Gyrus | 8 | -86 | -2 | 7.75 |
| R Lingual Gyrus | 10 | -88 | -4 | 7.69 |
| R Cuneus | 18 | -96 | 12 | 7.59 |
| L Calcarine Gyrus | -8 | -92 | -8 | 7.58 |
| R Superior Occipital Gyrus | 24 | -82 | 18 | 7.09 |
| R Middle Occipital Gyrus | 28 | -86 | 20 | 7.02 |
| L Lingual Gyrus | -20 | -94 | -14 | 6.44 |
| L Cuneus | -8 | -90 | 26 | 5.47 |
| R Postcentral Gyrus | 24 | -32 | 72 | 3.98 |
| R Precuneus | 10 | -50 | 74 | 3.96 |
| R Precentral Gyrus | 16 | -24 | 74 | 3.92 |
| R Paracentral Lobule | 6 | -24 | 76 | 3.68 |
| L Posterior-Medial Frontal | -6 | -12 | 76 | 3.66 |
| L Paracentral Lobule | -6 | -18 | 76 | 3.62 |
| L Heschls Gyrus | -42 | -16 | 6 | 4.47 |
| L Insula Lobe | -44 | -10 | 2 | 4.25 |
| L Rolandic Operculum | -44 | -6 | 2 | 4.20 |
| R Superior Temporal Gyrus | 54 | -4 | 2 | 4.10 |
| R Insula Lobe | 44 | -8 | 4 | 4.05 |
| R Heschls Gyrus | 46 | -20 | 6 | 3.57 |

**Figure S2.** Classification result of seed-regions EBA, LOC and FBA for different parameter values of *k* (number of nearest neighbours used for classification in knn-classification). Plots follow the same structure as in Figure 6. EBA, LOC and FBA were classified as either ‘dorsal’ or ‘ventral’ based on whole brain connectivity patterns of several dorsal and ventral stream areas (see Table 2; *Definition of dorsal and ventral stream target-regions*) for left (light grey) and right (dark grey) hemispheres. Parameter values for k range from 2 to 8 (i.e. one less than number of subregions in the ventral stream mask). Bars represent the probability of a seed region being classified as ‘dorsal’, ranging from 0 (always ventral) to 1 (always dorsal). Bars are centred to p=.5, i.e. equally likely to be classified as dorsal or ventral. Error bars indicate standard error of the mean. All statistics based on Friedman’s tests (df=30) comparing pairs of seed regions per hemisphere. * p < .05; ** p < .01; *** p < .001; n.s., not significant.

***
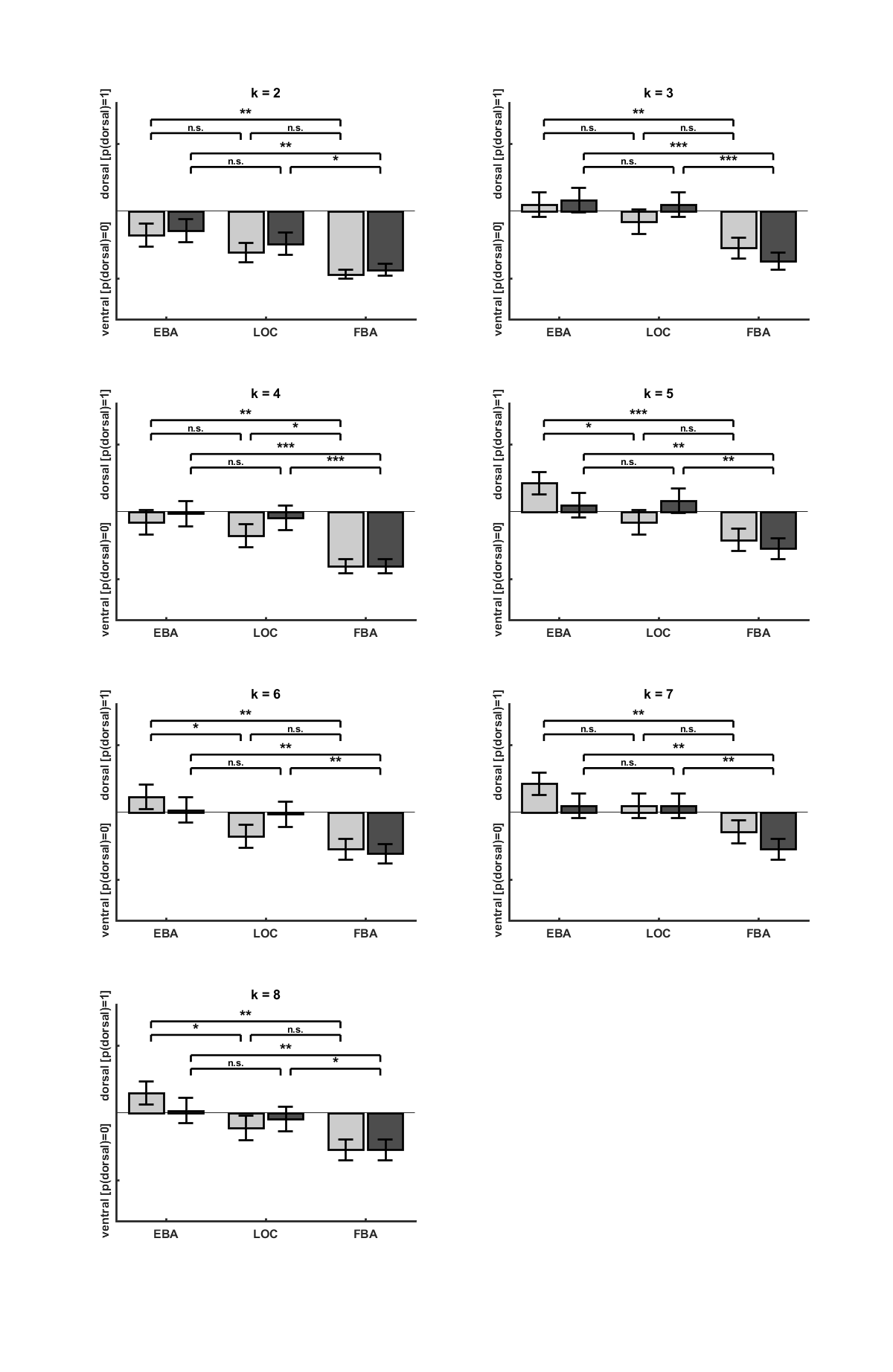
***

**Figure S3.** Functional (resting state) connectivity strength for all regions of the HCP-MMP1.0 atlas (Glasser et al. 2016) individually, for left (top) and right (bottom) hemispheres (see also Figure 5). Connectivity strength (beta values, see *ROI based functional connectivity*) of the seed regions (EBA, FBA, LOC) is shown for each region of the HCP-MMP1.0 atlas in pie charts. Pie charts consist of two parts: the right half shows positive correlations, the left half shows negative (anti-)correlations. For readability, square roots of beta-values are shown (increasing smaller values). Values are scaled such that the strongest connected HCP-MMP1.0 region (sum of all seed’s values) fills half a circle. Colored lines show label borders of the HCP-MMP1.0 atlas on a flat brain with sulcus map background; border colors correspond to HCP-MMP1.0 label colors.

***
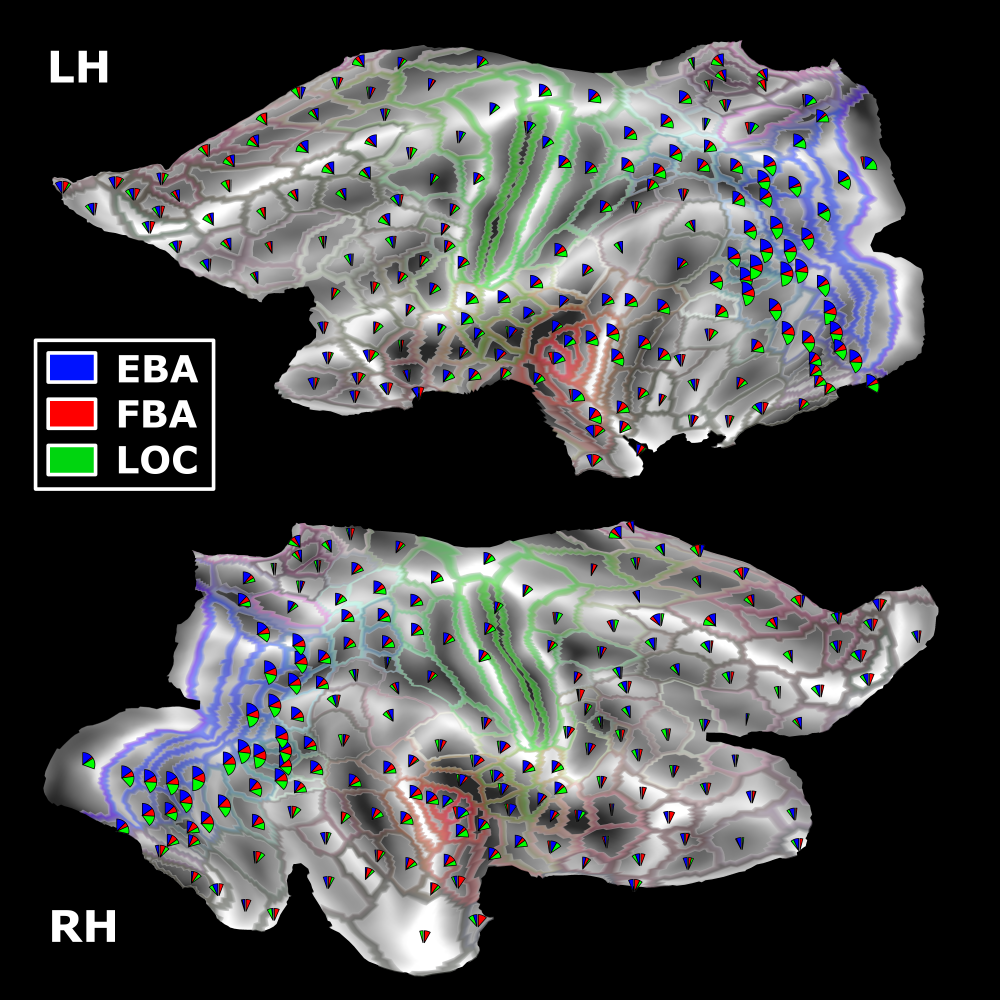
***

**Figure S4.** Probabilistic tractography of diffusion-weighted MRI for all regions of the HCP-MMP1.0 atlas (Glasser et al. 2016) individually, for left (top) and right (bottom) hemispheres (see also Figure 7). Connection probability (log transformed tract probability; see *Image processing and analysis of diffusion-weighted MRI*) of the seed regions (EBA, FBA, LOC) is shown for each region of the HCP-MMP1.0 atlas in pie charts. For readability, square roots of log transformed tract probabilities are shown (increasing smaller values). Values are scaled such that the strongest connected HCP-MMP1.0 region (sum of all seed’s values) scales to a full pie chart. Colored lines show label borders of the HCP-MMP1.0 atlas on a flat brain with sulcus map background; border colors correspond to HCP-MMP1.0 label colors.

***
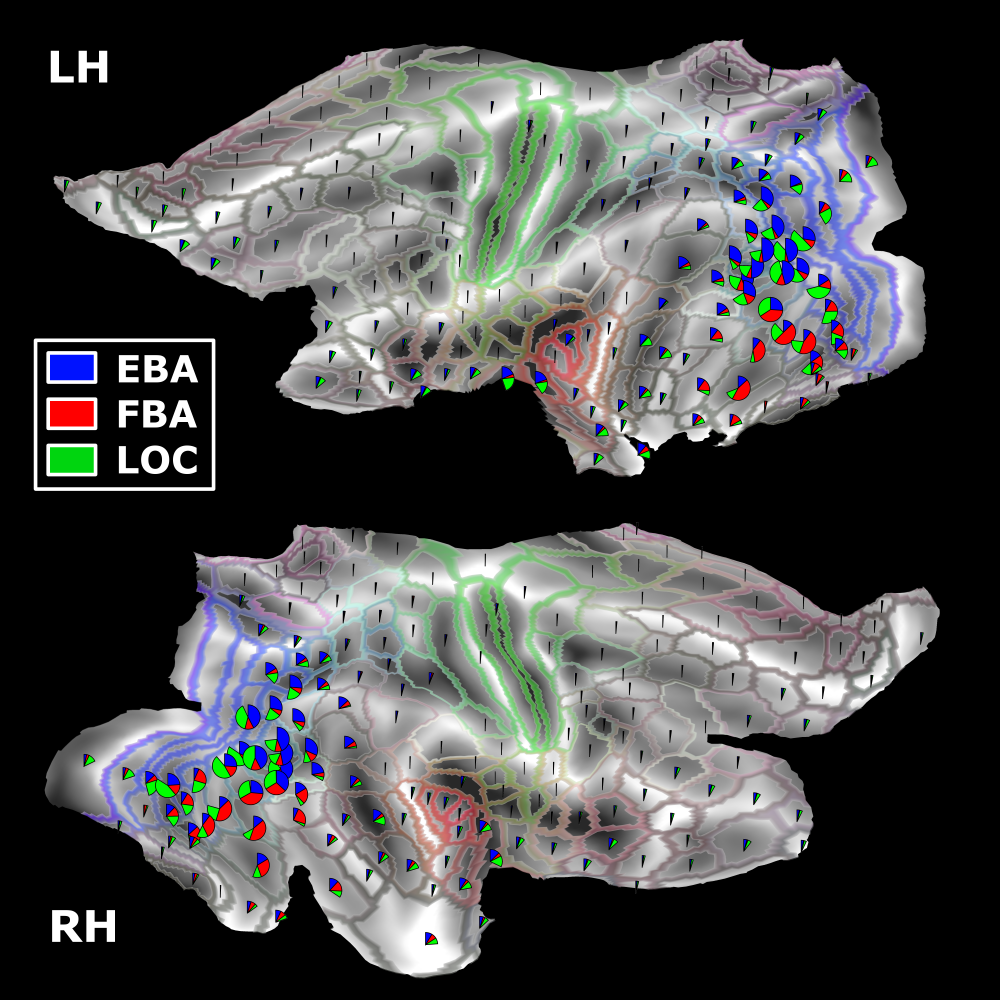
***
